# Distinct outcomes of CRL–Nedd8 pathway inhibition reveal cancer cell plasticity

**DOI:** 10.1038/cddis.2016.395

**Published:** 2016-12-01

**Authors:** Anastasia V Rulina, Frédérique Mittler, Patricia Obeid, Sophie Gerbaud, Laurent Guyon, Eric Sulpice, Frédérique Kermarrec, Nicole Assard, Monika E Dolega, Xavier Gidrol, Maxim Y Balakirev

**Affiliations:** 1 Commissariat a l'Energie Atomique et aux Energies Alternatives (CEA), Institut Biosciences and Biotechnology-Grenoble (BIG) BGE-BIOMICS, F-38054 Grenoble, France; 2 University Grenoble Alpes, BIG-BGE, F-38000 Grenoble, France; 3 INSERM, BGE, F-38000 Grenoble, France

## Abstract

Inhibition of protein degradation by blocking Cullin-RING E3 ligases (CRLs) is a new approach in cancer therapy though of unknown risk because CRL inhibition may stabilize both oncoproteins and tumor suppressors. Probing CRLs in prostate cancer cells revealed a remarkable plasticity of cells with TMPRSS2-ERG translocation. CRL suppression by chemical inhibition or knockdown of RING component RBX1 led to reversible G0/G1 cell cycle arrest that prevented cell apoptosis. Conversely, complete blocking of CRLs at a higher inhibitor dose-induced cytotoxicity that was amplified by knockdown of CRL regulator Cand1. We analyzed cell signaling to understand how varying degrees of CRL inhibition translated to distinct cell fates. Both tumor suppressor and oncogenic cell signaling pathways and transcriptional activities were affected, with pro-metastatic Wnt/*β*-catenin as the most upregulated. Suppression of the NF-*κ*B pathway contributed to anti-apoptotic effect, and androgen receptor (AR) and ERG played decisive, though opposite, roles: AR was involved in protective quiescence, whereas ERG promoted apoptosis. These data define AR–ERG interaction as a key plasticity and survival determinant in prostate cancer and suggest supplementary treatments that may overcome drug resistance mechanisms regulated by AR–ERG interaction.

The major goal of cancer therapy is to suppress malignant neoplasms without detriment to normal cells. Ubiquitin-proteasome system (UPS) has emerged as one of the principal cancer targets.^1^ In cancer cells, posttranslational downregulation of tumor suppressors involves proteasomal proteolysis making the cells particularly dependent upon UPS. Moreover, because of a significant degree of aneuploidy and rapid proliferation, cancer cells produce many abnormal proteins leading to permanent proteotoxic stress.^2^ As a result, inhibition of the proteasome is efficient against many types of cancer.

Beside the proteasome, other potential targets from UPS include cullin-RING E3 ligases (CRLs). Notably, inhibition of CRLs stabilizes a number of tumor suppressors without affecting global cellular catabolism, and seems to be more specific than targeting the proteasome.^3^ CRLs are multi-protein complexes assembled in mammals on seven cullin scaffolds (cullins 1, 2, 3, 4a, 4b, 5, and 7).^4^ Pro-degradative activity of CRLs requires cullin modification with a small ubiquitin-like protein Nedd8.^5^ Similar to ubiquitylation, neddylation involves an ordered transfer of Nedd8 by specific E1-activating enzyme (Nae1/Uba3 heterodimer NAE), E2-conjugating enzymes (Ube2F or Ube2M) and E3 ligases (Rbx1 and Rbx2 for CRLs, and others). Some of these enzymes are druggable providing a way to block CRL function.^6^ NAE inhibitor, MLN4924 (MLN), efficiently abrogates cullin neddylation and suppresses the growth of various types of cancer cells *in vitro* and *in vivo*.^3^ MLN is currently in clinical trials for the treatment of hematological malignancies and solid tumors.^7, 8^

Even with the growing evidence for non-cullin Nedd8 regulation,^5^ all proposed mechanisms of MLN action implicate CRL inhibition.^3, 9, 10, 11, 12, 13, 14, 15^ Despite the evident complexity of the CRL–Nedd8 network, only two main cellular responses have been documented, senescence and apoptosis, considerably fewer than might have been expected. The answer may lie in cell type-specific sensitivities to MLN. For example, despite a unique target (NAE, IC_50_ ~5 nM), MLN toxicity in various cell lines varied over three orders of magnitude showing that CRL suppression does not necessarily lead to cell death.^3^ Notably, recent studies have shown that CRL inhibition by MLN induces autophagy that protects cancer cells from apoptosis; moreover, blocking autophagy markedly enhanced drug efficacy.^16, 17^ Therefore, analysis of all possible effects of CRL inhibition is of clinical importance as it may lead to improved drug efficacy.

Herein, we provide new insights into CRL inhibition as a potential anti-cancer approach by elucidating its cancer-specific consequences in prostate cancer cells. We demonstrate that suppression of androgen receptor (AR), cullin-associated and neddylation-dissociated 1 protein (Cand1), and unknown PS1145 targets sensitize cancer cells to CRL inhibition. We also discuss potential pitfalls of this approach (resistant dormant state, activation of pro-metastatic pathways) that manifest the integrated nature of the cancer cell response and that must be taken into account in pre-clinical evaluation of CRL inhibitors.

## Results and Discussion

### Differential sensitivities of prostate cancer cell lines to MLN

**Figure 1:**
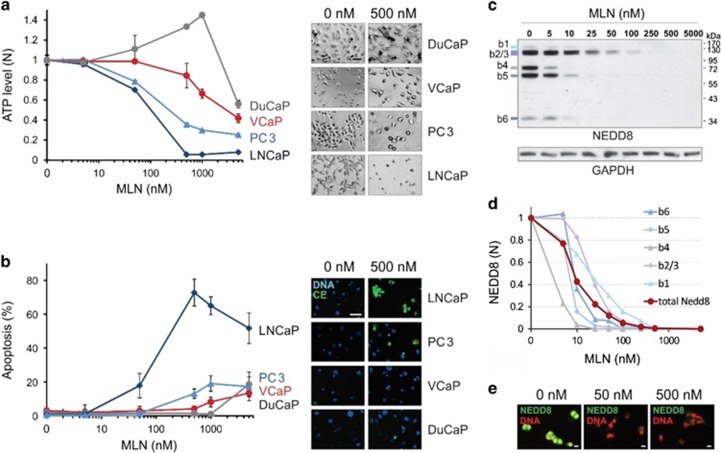
MLN effect on prostate cancer cell lines. (**a**) Plot of cellular ATP *versus* MLN concentration (mean±S.D.). Microscopy images on the right show the changes in cell morphology at 500 nM MLN. Scale bar, 100 *μ*m. (**b**) Apoptosis induction by MLN shown as percentage of apoptotic cells (mean±S.D.). Fluorescence microscopy images on the right show Hoechst (DNA, blue) and CellEvent (CE, green) staining. Scale bar, 50 *μ*m. (**c**) Analysis of Nedd8 conjugates in VCaP cells by western blotting with Nedd8-specific antibody and anti-GAPDH for loading control. The sizes of bands 1–6 correspond to neddylated cullins (b2/3), NAE1 (b4), UBA3 (b5), and Ube2M (b6). (**d**) The abundance of Nedd8 species was quantified by ImageJ using (**c**), and normalized first to GAPDH, then to vehicle control. (**e**) Immunofluorescence analysis of Nedd8 conjugates in VCaP cells with Nedd8-specific antibody (NEDD8, green) and Hoechst dye (DNA, red). Scale bar, 10 *μ*m

To investigate MLN potency against different types of prostate cancer, we examined its effect in LNCaP, PC3, DuCaP, and VCaP cancer cell lines. 3-day MLN treatment markedly decreased cellular ATP content in LNCaP cells, resulting in 95% cell mortality at 500 nM MLN (Figure 1a and Supplementary Figure S1). PC3 cells showed lower, but still significant, mortality and decline in ATP levels (>60% at 500 nM MLN). In contrast, DuCaP and VCaP cells were largely resistant to ⩽1 *μ*M of MLN, showing little effect on ATP levels or number of cells.

To investigate whether the observed death of prostate cancer cells was caused by apoptosis, we examined the activation of caspases 3/7 using CellEvent fluorogenic substrate (Figure 1b and Supplementary Figure S1). MLN induced massive apoptosis in LNCaP cells, while PC3 cells were less affected. Consistent with an MLN-resistant phenotype, VCaP and DuCaP cells showed negligible caspase activity, even at 1 *μ*M MLN (Figure 1b). Both VCaP and DuCaP cell lines contain amplified *AR* gene and upregulated ERG transcription factor that results from *TMPRSS2-ERG* (TER) chromosomal translocation.^18^ This particular genetic context may render VCaP and DuCaP cells less dependent on the CRL–Nedd8 pathway.

The observed MLN resistance of TER-positive cells could result from inefficient inhibition of NAE in these cell lines. However, the analysis of protein neddylation in VCaP cells revealed that the inhibition efficacy was quite similar to that of MLN-sensitive cell lines PC3 and LNCaP, attaining ~90% suppression at 50 nM and almost complete abrogation of neddylation at 100 nM MLN (Figures 1c–e, Supplementary Figures S2A and B). The inhibition was NAE specific, as no changes were observed for ubiquitin and SUMO conjugates (Supplementary Figure S2C).

VCaP and DuCaP cells have much longer doubling times than LNCaP and PC3 cells.^19^ As the toxic effect of MLN on cancer cells was shown to be proliferation-dependent,^11^ the slower growth of VCaP and DuCaP cells may contribute to their resistance; however, despite the different proliferation rates (Supplementary Figure S3), similar MLN dose–responses were observed in cells cultured on standard (Supplementary Figure S1) and androgen-deprived (Figure 1) media, suggesting that cell cycling was not the only factor determining MLN toxicity.

### Distinct outcomes of Nedd8 pathway inhibition

Extending MLN treatment of VCaP cells to 5 days increased cell apoptosis, albeit only for drug concentrations >500 nM (Supplementary Figure S4A). Curiously, for lower drug doses we observed a statistically significant decrease in spontaneous apoptosis.

**Figure 2:**
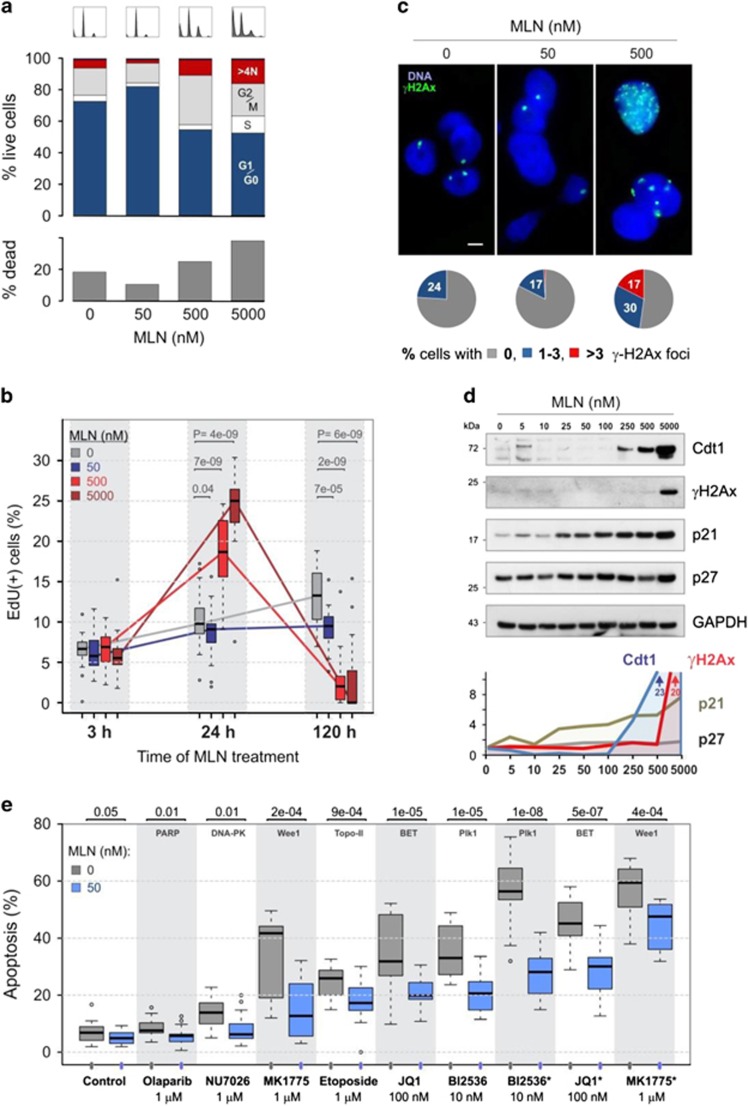
Distinct outcomes of MLN treatment. (**a**) Effect of MLN on VCaP cell cycle analyzed by flow cytometry (profiles are shown on the top). DNA content was measured with 7-AAD. The percentage of dead cells was estimated by counting cells with <2N DNA content. (**b**) Effect of MLN on cellular DNA synthesis measured by EdU incorporation. The percentage of EdU-positive cells is shown as a boxplot diagram (see also Supplementary Figure S4). (**c**) Immunofluorescence analysis of DNA breaks with *γ*-H2AX-specific antibody (*γ*-H2AX, green) and Hoechst dye (DNA, blue). Scale bar, 5 *μ*m. Diagrams show percentage of nuclei with an indicated number of *γ*-H2AX foci at 0 nM MLN (*n*=100), 50 nM MLN (*n*=125), and 500 nM MLN (*n*=115). (**d**) Effect of MLN on selected cellular markers. Western blotting was performed with protein-specific antibodies. The protein level was normalized first to GAPDH, then to vehicle control. (**e**) Suppression of drug cytotoxicity by 50 nM MLN. Cells were treated for 5 days with indicated drugs with (blue) or without (gray) 50 nM MLN in 10% ChSM or 10% StdM (asterisk). Data are presented as boxplot diagrams with *P*-values indicated at top. For drug description see Supplementary Table S5.

Cytotoxic effects of MLN have been linked to the accumulation of a number of CRL substrates such as Cdt1, p21, Wee1, which may provoke DNA re-replication and cell cycle arrest.^9, 10, 11, 12^ We therefore examined how NAE inhibition affects the cell cycle (Figure 2a). Consistent with the reduced apoptosis, the treatment of cells with 50 nM MLN decreased the percentage of sub-G1/G0 (dead) cells (Figure 2a, bottom). Unexpectedly, the cells were also accumulating in the G1/G0 phase indicating cell cycle arrest (Figure 2a, top). As VCaP cells express mutant p53-R248W that is unable to induce G1 arrest,^20^ other mechanisms seem to be involved. Higher doses of MLN increased the fraction of dead cells, of cells in G2/M (500 nM MLN) and in S (5 *μ*M MLN) phases, as well as cells with high (>4N) DNA content. These data are in agreement with the proposed mechanism of MLN action, which includes stabilization of Cdt1, DNA re-replication, cell cycle arrest at S/G2/M, and apoptosis.^9, 10^ Corroborating this conclusion, western blot analysis revealed an elevated level of Cdt1 starting at 250 nM MLN (Figure 2d).

To detect DNA re-replication, we measured cellular DNA synthesis using EdU assay (Figure 2b). Here again, distinct responses to low and high doses of MLN were observed both in androgen-deprived and standard media (Figure 2 and Supplementary Figure S4B). Specifically, the treatment with 50 nM MLN induced progressive inhibition of EdU incorporation to 90–65% of control values at 24–120 h. However, at concentrations ⩾500 nM the effect was biphasic: at 24 h EdU signal rose to 200–250% of control value, followed by complete cessation of DNA synthesis at 120 h. The initial increase in EdU coincided with Cdt1 accumulation suggesting that it reflects DNA re-replication and repair processes; conversely, the subsequent shutdown of DNA synthesis probably results from an inability to repair the inflicted damage.

To ascertain that MLN induced DNA damage, we examined the status of Ser-140 phosphorylated histone H2AX (*γ*-H2AX), a marker of DNA double-strand breaks. Western blot analysis revealed a massive accumulation of *γ*-H2AX in the cells treated with 5 *μ*M MLN, with a slight increase already detectable at 250–500 nM (Figure 2d). In parallel, immunofluorescence staining showed multiple *γ*-H2AX foci in cells treated with 500 nM MLN (Figure 2c). Similar dose–responses of *γ*-H2AX foci formation and caspase activation pointed to DNA damage as a primary trigger of apoptosis.

Consistent with the previous reports,^21, 22^ we observed a relatively high basal level of *γ*-H2Ax foci and spontaneous apoptosis in VCaP cells, reflecting DNA damage caused by ERG upregulation. Notably, at 50 nM, MLN slightly decreased the incidence of *γ*-H2AX foci, implying that the G0/G1 cell cycle arrest and reduced rate of DNA synthesis induced at this dose prevented spontaneous DNA breaks. This also suggests that DNA damage was not the cause of G0/G1 arrest. We found an increase in cyclin-dependent kinase inhibitors p21^Cip1^ and p27^Kip1^ even at 25 nM MLN that may have contributed to cell growth inhibition (Figure 2d). Notably, G0/G1 arrest did not lead to apoptosis but instead, protected cells. This protective effect became evident when we screened for drugs toxic to VCaP cells. Consistent with ongoing DNA damage, the cells appeared to be very sensitive to the inhibition of G2/M regulators Plk1 and Wee1,^20, 23^ whereas 50 nM MLN considerably suppressed the efficacy of chemotherapy (Figure 2e).

Collectively, these data suggest that, depending on the dose, MLN can instigate two distinct cell responses. At concentrations <100 nM (MLN^low^), the drug arrests cells in G1/G0 phase and inhibits DNA synthesis, thus preventing DNA damage and apoptosis; whereas at concentrations >250 nM (MLN^high^) MLN causes DNA damage, S/G2/M cell cycle arrest, and apoptosis.

### MLN induces reversible growth arrest in 3D spheroid model

**Figure 3:**
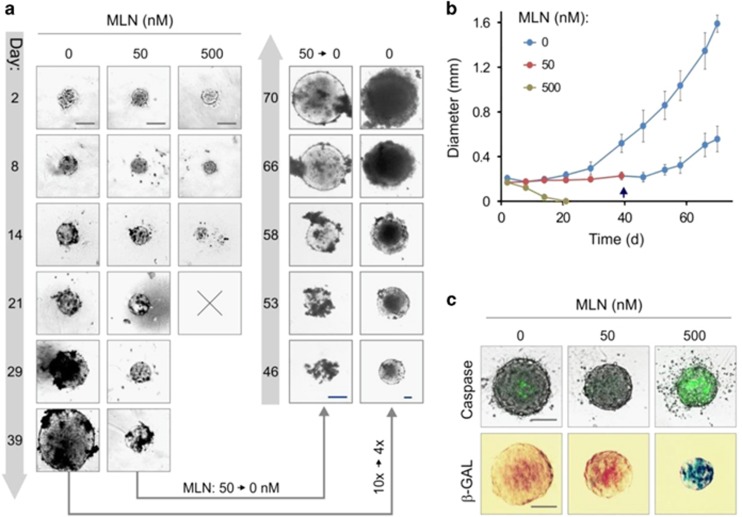
MLN induces reversible growth arrest in spheroid model. (**a**) Effect of MLN on spheroid growth analyzed by phase-contrast microscopy. An × 10 objective was used, except for control spheroids, for which starting from day 46, an × 4 objective was used. On day 40, spheroids cultured with 50 nM MLN were transferred into MLN-free medium. Scale bars, 200 *μ*m. (**b**) Quantitative analysis of spheroid growth based on microscopy images (mean±S.D.). (**c**) Induction of apoptosis and senescence by MLN. Top phase-contrast/fluorescent microscopy images show Caspase-3/7 activity measured with CellEvent. Bottom panels show senescence-associated beta-galactosidase activity (*β*-GAL) measured with X-GAL. Scale bar, 100 *μ*m. See also Supplementary Figures S5–S8

The above results suggest that the resistance of VCaP cells to MLN-induced apoptosis came from cell cycle arrest in G0/G1 phase. The principal question relating to the potential clinical application of MLN is whether this arrest is reversible as it may lead to tumor regrowth. To address this, we investigated NAE inhibition in a tumor-relevant 3D spheroid model (Figure 3). Exponential spheroid growth was observed in the control condition, while 50 nM MLN blocked growth of spheroids for 10 weeks without visible impact on their integrity (Figures 3a, b and Supplementary Figure S5). In contrast, at 500 nM, MLN caused complete dissolution of the spheroids within 2–3 weeks, corroborating our findings from 2D cell culture, where significant apoptosis was detected with MLN^high^ (Figure 1b and Supplementary Figure S4A). Confirming apoptotic cell death, strong caspase activation was detected at 500 nM MLN (Figure 3c and Supplementary Figure S6), whereas rare apoptotic events were found at 50 nM drug.

In some cell types MLN can trigger p21^Cip1^-dependent senescence.^10, 12^ As we had observed an increase in p21^Cip1^, we examined the activity of senescence-associated beta-galactosidase (SA-*β*-GAL). Negligible activity was detected in control and 50 nM MLN conditions, while spheroids treated with 500 nM MLN showed intense SA-*β*-GAL staining (Figure 3c and Supplementary Figure S7). We concluded that the cell growth arrest imposed by MLN^low^ is not due to senescence and may be reversible. Indeed, transferring spheroids arrested for 40 days into drug-free medium resulted in spheroid regrowth at a pace similar to that of control (Figures 3a, b and Supplementary Figure S8).

Collectively, these results demonstrate that, depending on the dose, MLN treatment can have completely different outcomes, reversible quiescence (MLN^low^) or apoptosis (MLN^high^). These data further confirm the existence of a protective dormant state that should be taken into account in clinical applications of MLN.

### Knockdown of CRL components results in opposite effects on cell proliferation and survival

**Figure 4:**
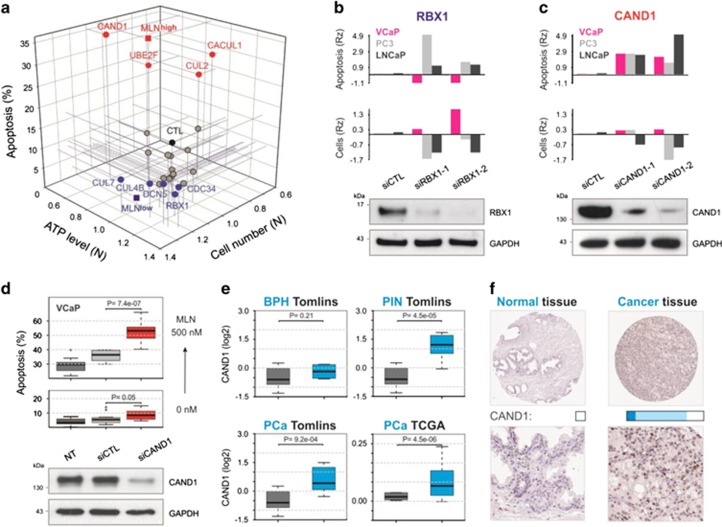
Distinct roles of CRL components in cell regulation. (**a**) Effect of gene knockdown on VCaP cell proliferation and survival was measured after 5 days of siRNA treatment and compared with control siRNA (siCTL) and 50 and 500 nM MLN (MLN^low^ and MLN^high^). Cell number and ATP level were normalized to the values of siCTL-treated cells. The major ‘hits' are shown (for all genes, see Supplementary Figure S9). (**b**) Survival of prostate cancer cells after *RBX1* knockdown with two individual siRNAs. Statistical robust Z-score was used (RZ, see Supplementary Information). Knockdown efficacy was confirmed by western blotting with Rbx1-specific antibody. (**c**) Survival of prostate cancer cells upon *CAND1* knockdown with two individual siRNAs. Analysis as in **b**. (**d**) Effect of *CAND1* knockdown on MLN-induced apoptosis. VCaP cells were transfected with 5 nM of *CAND1* siRNA or with the same amount of siCTL and treated with MLN (see also Supplementary Figure S10). (**e**) *CAND1* transcripts are upregulated in prostate cancer. Expression of *CAND1* in benign prostatic hyperplasia (BHP, blue), prostatic intraepithelial neoplasia (PIN, blue), and prostate cancer (PCa, blue) in comparison with normal prostatic tissue (gray) (Oncomine database,^27^ www.oncomine.org). (**f**) Immunohistochemical staining for Cand1 in prostate tumors and normal tissues (Human Protein Atlas,^28^ www.proteinatlas.org)

The discontinuous effect of MLN on cell fate is not entirely unexpected. CRLs, which are the major neddylation substrates,^5^ regulate hundreds of cellular factors with distinct impacts on cell function. Plausibly, the inhibition of some CRLs would promote cell death, while the inhibition of others would favor pro-survival processes. Thus, the outcome would be discontinuous depending on the role, weight, and sensitivity of each component within the CRL network. To test this hypothesis, we screened a set of 22 siRNAs targeting principal components of the CRL–Nedd8 system (Supplementary Table S2). The changes in cell number and apoptosis were monitored by automated microscopy and cellular ATP level was measured separately (Figure 4a and Supplementary Figure S9). Gene knockdowns were compared with 50 and 500 nM doses of MLN.

We found that knockdown of *CUL1* and, particularly, *CUL2* inhibited cell growth and induced apoptosis, whereas knockdown of *CUL7* and *CUL4B* had the inverse effect, increasing cell number and/or reducing apoptotic rate. Several other CRL–Nedd8 components were also found on opposite ends: *CAND1*, *UBE2F*, *CACUL1* (MLN^high^-like, pro-apoptotic siRNAs) and *RBX1*, *DCN4*, *DCN5* (MLN^low^-like, cytoprotective siRNAs). Notably, knockdown of *RBX1*, a major E3-RING component of CRLs, phenocopied the effect of MLN^low^. Moreover, as had been the case with MLN, the cytoprotective effect of *RBX1* siRNAs was VCaP-specific: in ‘MLN-sensitive' LNCaP and PC3 cells, knockdown of *RBX1* increased apoptosis and decreased the number of cells (Figure 4b). Consistent with these observations, published studies have shown the same dichotomy: in some cases, *RBX1* knockdown induced apoptosis and senescence,^24^ whereas in others, acquisition of a drug-resistant phenotype.^25^ Hence, the particular outcome of NAE inhibition observed in VCaP cells was caused primary by CRL suppression.

Another prominent hit was *CAND1*, one of the key regulators of CRL balance. Cand1 acts as an exchange factor catalyzing redistribution of substrate receptors between CRLs.^26^ Knocking down *CAND1* induced significant apoptosis in all prostatic cell lines suggesting that Cand1 is a limiting component of the CRL network (Figures 4a, c and Supplementary Figure S9). Notably, there was a negative correlation between the level of Cand1 and the sensitivity of a cell line to MLN (Supplementary Figures S10A and B). We reasoned that if the MLN^high^ cytotoxicity was mainly due to compromised CRL function and was counteracted by Cand1, the inhibition of Cand1 would further potentiate the observed toxic effect. To examine this possibility, *CAND1* was suppressed with a suboptimal 5 nM concentration of siRNA resulting in the partial protein extinction and limited cell death (Figure 4d and Supplementary Figure S10C). Yet, this amount of siRNA significantly increased apoptosis induced by MLN^high^ pointing to an epistatic relationship between *CAND1* and *NAE*.

Taken together, these data (1) provide explanations for the differential effect of MLN in VCaP cells; (2) support the role of CRLs as a major effector of NAE inhibition; (3) put forward Cand1 as a potential therapeutic target in prostate cancer. In support of the latter, interrogation of the Oncomine database retrieved *CAND1* in the top 2% of upregulated genes in prostate intraepithelial neoplasia (PIN), and in the top 3% percent in prostate adenocarcinoma^27^ (Figure 4e). Cand1 upregulation in prostate cancer is also supported by data from the Human Protein Atlas^28^ (Figure 4f).

### CRL inhibition globally affects cell signaling and transcription

**Figure 5:**
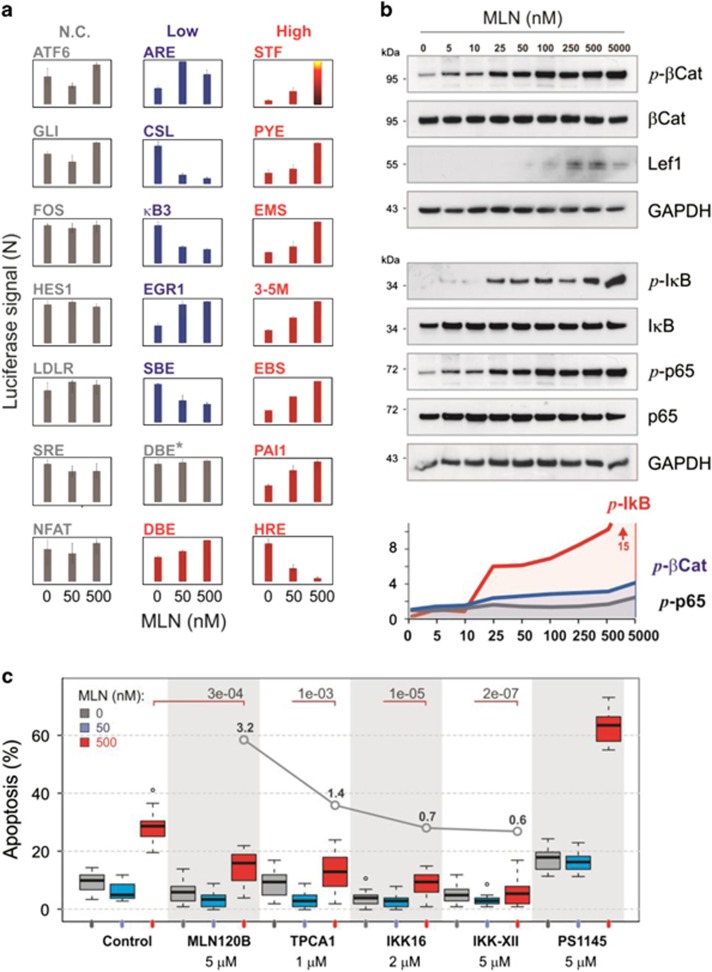
Inhibition of CRL–Nedd8 pathway globally affects cell signaling and transcription. (**a**) Dual luciferase reporter assay with VCaP cells co-transfected with control Renilla luciferase and Firefly luciferase reporter vectors (Table 1 and Supplementary Table S3). Firefly luciferase signal was first normalized to Renilla luciferase, and then to vehicle control (mean±S.D.). Responses were divided into; non-classified (NC, see Table 1); MLN^low^- specific (low); MLN^high^- specific (high). (**b**) MLN stabilizes cell signaling factors. Western blotting was performed with protein-specific antibodies. The protein level normalized first to GAPDH, then to vehicle control. (**c**) Effect of IKK inhibitors on MLN-induced apoptosis. Cells were treated for 5 d with indicated drugs in 10% ChSM. The data are presented as a boxplot diagram with *P*-values for 500 nM MLN. The four-point plot inside the diagram shows IKK*β*-to-IKK*α* selectivity of the drugs.^34^ See also Supplementary Figures S11–S13

The above findings suggest that the distinct outcomes observed for MLN treatment were due to stabilization of the CRL substrates implicated in the cell fate decision. By using a panel of luciferase reporters, we examined the effect of MLN on the activity of major signaling and transcriptional regulators (Table 1 and Supplementary Table S3). The majority of the examined pathways were affected: one subset led to cell cycle arrest and included NF-*κ*B inhibition, EGR1 and FoxO stimulation, and so on, whereas another promoted cell proliferation and invasiveness and included upregulation of ERG, cMyc, Wnt/*β*-catenin (Wnt/*β*-Cat), and so on. The dose-response varied depending on the specific promoter that further explains the discontinuous effect observed with MLN^low^ and MLN^high^. To identify pathways potentially responsible for these outcomes, we tentatively classified the reporter responses as MLN^low^-specific (>80% maximal effect observed at 50 nM MLN), and MLN^high^-specific (significant change observed between 50 and 500 nM MLN; Table 1 and Figure 5a). Notably, cell cycle arrest-promoting effects appeared mainly as MLN^low^-specific, whereas the majority of those favoring proliferation were assigned to the MLN^high^ group (Table 1).

The most striking response was observed with Wnt/*β*-Cat reporter (STF, ~190-fold increase at 500 nM MLN, Table 1). This is likely due to the inhibition of degradation of signaling-engaged *β*-Cat.^29^ Indeed, MLN stabilized phospho-*β*-Cat and induced its accumulation at the nuclear membrane (Figure 5b and Supplementary Figure S11A). We also observed an increase in the transcription factor Lef1, a *β*-Cat-partner, that was probably induced as a result of upregulated ERG,^30^ also classified in the MLN^high^-group (PYE and EBS reporters, Table 1). Notably, the hyperactivation of *β*-Cat/Lef1 signaling did not contribute to MLN^high^ cytotoxicity, as the inhibitors of Wnt/*β*-Cat-pathway had only a minor effect on apoptosis (Supplementary Figure S11B).

Also remarkable was a complete inhibition of hypoxia-responsive HRE reporter. This reporter displayed a high background signal indicating constitutive activation of hypoxia-inducible factor 1-alpha (HIF-1*α*) in VCaP cells. As the CRL1^Skp2^ substrate FoxO3a counteracts HIF-1*α* activity on the HRE promoter,^31^ stabilization of FoxO3a may explain the observed inhibition. Consistent with this notion, MLN also stimulated the activity of FoxO-dependent DBE reporter.

The suppression of HRE reporter by MLN and the absence of the effect on mTOR signaling (LDLR and SRE reporters, Figure 5a and Table 1) argue against HIF-1*α* -dependent inhibition of mTORC1 pathway and subsequent autophagy stimulation.^17^ The latter was also not supported by the examination of autophagy markers and of the effect of autophagy inhibitors on VCaP apoptotic response (Supplementary Figure S12).

Among the MLN^low^-specific responses, one of the most prominent was the inhibition of NF-*κ*B signaling (*κ*B3, Table 1 and Figure 5a). NF-*κ*B is a MLN target in activated B cell-like diffuse large B-cell lymphoma, where its inhibition induces G1 cell cycle arrest and apoptosis.^13^ NF-*κ*B also plays an important role in prostate carcinogenesis, and particularly in ETS-positive cancers, where it is an ERG target.^32, 33^ We found a marked buildup of phosphorylated NF-*κ*B inhibitor I*κ*B (p-I*κ*B), the likely cause of the observed NF-*κ*B inhibition (Figures 5a and b). On the other hand, MLN also increased the level of phospho-p65-Ser536, a major active ERG-dependent form of NF-*κ*B in VCaP cells.^33^ However, the phospho-p65-Ser536 accumulated within cytoplasmic speckles, and, thus, was transcriptionally inactive (Supplementary Figure S13A). The suppression of NF-*κ*B signaling in VCaP cells has been shown to reduce cell growth.^33^ As the MLN dose-response of the *κ*B3 reporter correlated with the MLN^low^ phenotype, NF-*κ*B inhibition may contribute to cell cycle arrest and the anti-apoptotic effect produced by MLN^low^. To address this question we examined the level of MLN-induced apoptosis after blocking the NF-*κ*B pathway with I*κ*B kinase (IKK) inhibitors. Among five different compounds tested, four significantly decreased apoptosis (Figure 5c and Supplementary Figure S13B). The protective effect was stronger with more promiscuous IKK inhibitors (with lower IKK*β*-to-IKK*α* selectivity,^34^ Supplementary Table S5) that affect not only the I*κ*B-dependent canonical pathway, a target of MLN, but also non-canonical IKK*α*-dependent NF-*κ*B signaling ^32^ (Figure 5c and Supplementary Figure S13B). Curiously, one of the inhibitors, PS1145, strongly stimulated MLN cytotoxicity (Figure 5c). As its more specific structural analog, MLN120B^34, 35^ had the opposite effect, this was most likely due to the off-target inhibition of a non-IKK protein kinase,^35^ the identification of which may provide a complementary target for MLN treatment.

These results demonstrate that CRL inhibition globally changes cell signaling. Notably, both tumor suppressor and pro-oncogenic pathways are affected. Cell fate decision is, therefore, a result of the integration of multiple antagonistic events triggered by MLN. Specifically, the suppression of the NF-*κ*B pathway counteracts apoptosis and, thus, contributes to MLN^low^ phenotype, whereas the stimulation of ERG/cMyc-dependent signaling may promote proliferation-dependent MLN^high^ toxicity. Of clinical importance, MLN hyperactivates Wnt/*β*-Cat signaling that, in combination with upregulated FoxOs, may be a potent metastasis inducer.^36^

### Opposite roles of androgen receptor and ERG

G0/G1 cell cycle arrest and exit into the quiescent state is an essential step in the differentiation process. In normal prostate and low-grade primary tumors this process is AR-dependent.^37^ AR can also inhibit proliferation of cultured prostate cells.^38, 39, 40^ Because MLN stimulated ARE reporter (Table 1 and Figure 5a), we asked whether cell cycle arrest induced by MLN^low^ is accompanied by an activation of the AR transcriptional program.

**Figure 6:**
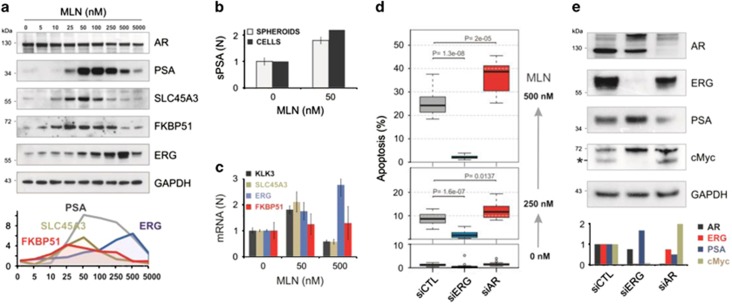
Opposite roles of AR and ERG in MLN-induced cell fate. (**a**) Effect of MLN on AR and AR-dependent proteins. Western blotting was performed with protein-specific antibodies. The protein level was normalized first to GAPDH, then to vehicle control. (**b**) MLN^low^ stimulates the secretion of PSA by VCaP cells and spheroids as assessed by ELISA. (**c**) Effect of MLN on the expression of AR target genes measured by quantitative RT-PCR (Supplementary Table S4). Data are normalized to 18 S rRNA expression and to vehicle control (mean±S.D.). (**d**) Opposite effects of AR and ERG knockdowns on MLN-induced apoptosis. VCaP cells were transfected with indicated siRNAs and treated with MLN (see also Supplementary Figure S14). (**e**) Changes in protein expression induced by *AR* and *ERG* knockdowns analyzed by Western blotting with protein-specific antibodies. Asterisk indicates active c-Myc isoform. The protein level was normalized first to GAPDH, then to siCTL

First, we analyzed the effect of MLN on the protein levels of AR, and the AR-specific differentiation markers: PSA, SLC45A3, and FKBP51 (Figure 6a). MLN did not significantly change the amount of AR. Strikingly, the drug induced a several-fold increase in the PSA level, with a maximum effect at 50 nM and decreasing levels at higher doses. Similar bell-shaped dose–response curves were observed for SLC45A3 and FKBP51 indicating AR activation. In parallel, we found that cells and spheroids arrested by MLN in G0/G1 secreted twice more PSA than untreated controls (Figure 6b). To examine if the changes seen on the protein level were due to variations in gene expression the corresponding transcripts were measured by quantitative RT-PCR (Figure 6c). Consistent with the western blot results, 50 nM MLN stimulated the expression of the target genes, while at 500 nM the transcripts were suppressed.

In TER-positive prostate cancer cells ERG expression is regulated by AR.^18^ The dose–response of ERG was, however, different from that of other AR targets, showing progressive increase in protein and transcript levels up to 500 nM MLN (Figures 6a and c). Two recent reports have demonstrated that degradation of ERG protein is controlled by CRL3^SPOP^ ligase,^41, 42^ findings that we initially thought explained the MLN-induced accumulation of ERG. However, MLN mainly upregulated shorter N-truncated versions of ERG (T1E4),^43^ which are poor CRL3^SPOP^ substrates;^41, 42^ furthermore, MLN did not increase the level of AR, another substrate of CRL3^SPOP^.^44, 45^ Finally, all examined CRL substrates reached maximum levels at 5 *μ*M MLN, at which the level of ERG had already dropped (Figure 6a). Thus, the decrease in the ERG level is more consistent with regulation at the transcriptional level.

It has been shown that ERG binding to AR and AR target genes disrupts androgen signaling.^46^ Our finding that MLN^low^ activates AR-dependent transcription implies that it relieves AR from ERG suppression. This may switch the cellular program from a potentially detrimental pro-proliferating regime to a differentiation-like quiescent state, thus protecting cells from re-replication-inflicted DNA damage. To the contrary, as MLN^high^ induces ERG and suppresses AR (Figures 5a, 6a and c), it promotes cell cycling and DNA damage. Thus the cytotoxic effect of MLN may be potentiated by ERG and counteracted by AR. To test this hypothesis, we examined the effect of AR and ERG knockdowns on MLN-induced apoptosis. Knockdown of ERG strongly suppressed the levels of ERG and c-Myc protein, a key mediator of ERG-dependent transformation^47^ (Figure 6e). This was accompanied by an increase in the PSA level consistent with AR de-repression.^46^ Strikingly, ERG knockdown strongly suppressed MLN-induced apoptosis (Figure 6d and Supplementary Figure S14). This suppression may be, partially due to AR activation as the stimulation of AR by dihydrotestosterone also had an anti-apoptotic effect (Supplementary Figure S14C). In contrast, AR knockdown significantly increased MLN cytotoxicity throughout the whole range of drug concentrations.

These data support an antagonistic role of AR and ERG in response to MLN, and demonstrate that the cell cycle arrest induced by MLN^low^ is associated with AR activation and expression of prostate differentiation markers, while MLN^high^ induces ERG/c-Myc resulting in proliferation-dependent apoptosis. Studies have shown that high androgen doses can reverse oncogenic AR transformation and suppress the growth of cancer cells.^48^ Herein, we demonstrated that the same result can be achieved by subtotal inhibition of CRLs. However, although AR re-activation by MLN does suppress cancer cell growth, its potential clinical benefit is uncertain as this effect remains reversible, and protects cancer cells from apoptosis.

## Materials and methods

Complete experimental details are given within the Supplementary Information.

### Cell culture

All cell lines were purchased from the American Type Culture Collection (ATCC, Manassas, VA, USA) except DuCaP, kindly provided by Jack Schalken (originally from Kenneth J Pienta^49^).

### ATP assay

To analyze cell metabolism, we measured ATP content with ViaLight Plus BioAssay according to the manufacturer's instructions (Lonza, Basel, Switzerland).

### Apoptosis assay

Apoptosis was assayed using CellEvent Caspase-3/7 Green Reagent (CE; Thermo Fisher Scientific, Villebon-sur-Yvette, France). Treatments were performed 1 d after cell seeding and CE was added at that time. Hoechst dye was added at the end of treatment. The image acquisitions were performed using CellInsight NXT High Content Screening Platform (Thermo Fisher Scientific). Hoechst staining was used for nuclear segmentation. Cells containing CE-positive nuclei were scored as apoptotic.

### DNA synthesis assay by EdU incorporation

DNA synthesis was measured using Click-iT EdU Alexa Fluor 647 Assay (Invitrogen). Treatments were performed 1 d after cell seeding. At specific times, the cells were treated with EdU for 5 h, then fixed and stained. Images were acquired on the CellInsight Platform. After nuclear segmentation with Hoechst, nuclear EdU signal was quantified.

### Cell cycle analysis

The ethanol-fixed cells were stained with 7-Aminoactinomycin D (7-AAD) and total DNA content per cell was measured on the BD LSR II flow cytometer (BD Biosciences, Bourgoin Jallieu, France).

### Senescence test

Senescence analysis was performed by measuring *β*-galactosidase activity. Spheroids were embedded into 1.5% low-melting agarose and fixed with 2% formaldehyde. Staining was performed with 1 mg/ml X-GAL, and viewed by bright field microscopy.

### Western blotting and ELISA

Standard procedures were used for western blotting. Secreted PSA was analyzed with Anogen Free PSA ELISA Kit. The antibodies are listed in Supplementary Table S1.

### siRNA transfection

Cell transfection with siRNA was performed with Lipofectamine RNAiMAX reagent (Invitrogen). CRL genes were screened using siRNAs from ON-TARGETplus SMART pool siRNA Library–Human Ubiquitin Conjugation Subset 1, complemented with ON-TARGETplus SMART pool siRNAs (Dharmacon) for *RBX1* and *SAG (RBX2).* Controls were: AllStars Negative Control siRNA and AllStars Hs Cell Death siRNA Positive cell death phenotype control (Qiagen). The sequences of siERG (Eurogentec) were as described.^50^ siRNA sequences are listed in Supplementary Table S2.

### Luciferase reporter assays

Luciferase measurements were done with the Dual-Luciferase Reporter Assay on GloMax-Multi Detection System according to the manufacturer's instructions (Promega, Charbonnières-les-Bains, France). The ratio of Firefly- to Renilla-luciferase activities was calculated. All values were presented as means±S.D. Reporter plasmids are listed in Supplementary Table S3.

### qPCR

Quantitative PCR (qPCR) was carried out with SuperMIX-UDG Kit (Thermo Fisher Scientific) on StepOnePlus Real-Time PCR system (Thermo Fisher Scientific). All experiments were run in triplicate, and results were normalized to 18 S rRNA expression. Primer sequences are listed in Supplementary Table S4.

## Figures and Tables

**Table 1:** CRL inhibition globally affects cell signaling and transcription

				**Fold change**^a^				**% Max**^b^	**MLN group**^c^
**Acronym**	**Reporter**	**Response elements**	**Pathway**	**0 nM**	**50 nM**	**500 nM**	**Effect**^d^	**50 nM**	
ARE	TAT-GRE-EIB	Androgen/glucocorticoid RE	AR/GR	1.00	2.70	1.87	↑	100.0	Low
CSL	4 × CSL	RBP-Jk binding site	Notch	1.00	0.24	0.15	↓	89.56	Low
κB3	κB3	NF-κB binding site	NF-*κ*B	1.00	0.44	0.37	↓	88.74	Low
EGR1	EGR1	EGR1 promoter	c-Myc/MAPK/others	1.00	2.21	2.38	↑	87.31	Low
SBE	12 × SBE	Smad-binding element	TGF-*β*/others	1.00	0.58	0.49	↓	81.37	Low
HRE	3 × HRE	Hypoxia response element	Hypoxia/FoxO/others	1.00	0.35	0.09	↓	71.49	High
PAI1	PAI1	PAI1 promoter	TGF-*β*/others	1.00	1.89	2.41	↑	63.28	High
EBS	6 × ETS	ETS-binding site	ERG	1.00	2.11	3.35	↑	47.39	High
3–5M	3′+5′ Myc	5′ plus 3′ c-MYC enhancer	Wnt/*β*-Cat/others	1.00	1.95	3.18	↑	43.88	High
DBE	3 × DBE	FoxO binding site	FoxO	1.00	1.24	1.68	↑	35.58	High
EMS	4 × EMS	E-box Myc sequence	c-Myc	1.00	1.50	3.68	↑	18.64	High
PYE	4 × Pye	Py enhancer element	ERG	1.00	1.34	3.61	↑	13.27	High
STF	14 × STF	Super TOP-flash, TCF/Lef1	Wnt/*β*-Cat	1.00	3.18	190.3	↑	1.14	High
DBE*	3 × DBE*	FoxO mut-binding site	FoxO-control	1.00	1.04	1.08	─	─	NC
ATF6	5 × ATF6	ATF6-binding site	ER-stress	1.00	0.66	1.42	↓↑	─	NC
GLI	8 × Gli	Gli-binding site	Hedgehog	1.00	0.75	1.38	↓↑	─	NC
FOS	FOS	c-fos promoter	MAPK/cAMP/others	1.00	0.93	1.01	─	─	NC
HES1	HES1	HES1 promoter	Notch/others	1.00	1.07	0.95	─	─	NC
LDRL	LDLR	LDLR promoter (LDLRp)	Akt/mTOR/others	1.00	1.26	1.17	─	─	NC
SRE	3 × (2-3LDLR)	Repeats 2 and 3 of the LDLRp	Akt/mTOR/others	1.00	0.80	0.81	─	─	NC
NFAT	3 × NFAT/AP1	NFAT/AP1-binding site	NFAT/AP1	1.00	0.88	1.20	─	─	NC
CTL	Control SV40	SV40 promoter	Control	1.00	0.91	0.95	─	─	NC

Firefly luciferase signal was normalized to Renilla luciferase in the same sample.

a Fold change compared with vehicle control (Flu_XnM_/Flu_0nM_).

b %Max_50nM_ was calculated as (ΔFlu_50nM_/ΔFlu_max_) × 100, where ΔFlu_50nM_=(Flu_50nM_ – Flu_0nM_), and ΔFlu_max_ is the maximal observed change.

c Response with %max_50nM_>80% was defined as MLN^low^-specific (low); and<80% as MLN^high^-specific (high); and NC, not classified.

d Stimulation (↑) and inhibition (↓).*- negative DBE control with mutated FoxO binding site

## References

[bib1] Hoeller D, Dikic I. Targeting the ubiquitin system in cancer therapy. Nature 2009; 458: 438–444. 19325623 10.1038/nature07960

[bib2] Luo J, Solimini NL, Elledge SJ. Principles of cancer therapy: oncogene and non-oncogene addiction. Cell 2009; 136: 823–837. 19269363 10.1016/j.cell.2009.02.024 PMC2894612

[bib3] Soucy TA, Smith PG, Milhollen MA, Berger AJ, Gavin JM, Adhikari S et al. An inhibitor of NEDD8-activating enzyme as a new approach to treat cancer. Nature 2009; 458: 732–736. 19360080 10.1038/nature07884

[bib4] Lydeard JR, Schulman BA, Harper JW. Building and remodelling Cullin-RING E3 ubiquitin ligases. EMBO Rep 2013; 14: 1050–1061. 24232186 10.1038/embor.2013.173 PMC3849489

[bib5] Enchev RI, Schulman BA, Peter M. Protein neddylation: beyond cullin-RING ligases. Nat Rev Mol Cell Biol 2015; 16: 30–44. 25531226 10.1038/nrm3919 PMC5131867

[bib6] Zhao Y, Morgan MA, Sun Y. Targeting neddylation pathways to inactivate cullin-RING ligases for anticancer therapy. Antioxid Redox Signal 2014; 21: 2383–2400. 24410571 10.1089/ars.2013.5795 PMC4241876

[bib7] Sarantopoulos J, Shapiro GI, Cohen RB, Clark JW, Kauh JS, Weiss GJ et al. Phase I study of the investigational NEDD8-activating enzyme inhibitor pevonedistat (TAK-924/MLN4924) in patients with advanced solid tumors. Clin Cancer Res 2016; 22: 847–857. 26423795 10.1158/1078-0432.CCR-15-1338

[bib8] Shah JJ, Jakubowiak AJ, O'Connor OA, Orlowski RZ, Harvey RD, Smith MR et al. Phase I study of the novel investigational NEDD8-activating enzyme inhibitor pevonedistat (MLN4924) in patients with relapsed/refractory multiple myeloma or lymphoma. Clin Cancer Res 2016; 22: 34–43. 26561559 10.1158/1078-0432.CCR-15-1237 PMC5694347

[bib9] Mackintosh C, Garcia-Dominguez DJ, Ordonez JL, Ginel-Picardo A, Smith PG, Sacristan MP et al. WEE1 accumulation and deregulation of S-phase proteins mediate MLN4924 potent inhibitory effect on Ewing sarcoma cells. Oncogene 2013; 32: 1441–1451. 22641220 10.1038/onc.2012.153

[bib10] Lin JJ, Milhollen MA, Smith PG, Narayanan U, Dutta A. NEDD8-targeting drug MLN4924 elicits DNA rereplication by stabilizing Cdt1 in S phase, triggering checkpoint activation, apoptosis, and senescence in cancer cells. Cancer Res 2010; 70: 10310–10320. 21159650 10.1158/0008-5472.CAN-10-2062 PMC3059213

[bib11] Milhollen MA, Narayanan U, Soucy TA, Veiby PO, Smith PG, Amidon B. Inhibition of NEDD8-activating enzyme induces rereplication and apoptosis in human tumor cells consistent with deregulating CDT1 turnover. Cancer Res 2011; 71: 3042–3051. 21487042 10.1158/0008-5472.CAN-10-2122

[bib12] Jia L, Li H, Sun Y. Induction of p21-dependent senescence by an NAE inhibitor, MLN4924, as a mechanism of growth suppression. Neoplasia 2011; 13: 561–569. 21677879 10.1593/neo.11420 PMC3114249

[bib13] Milhollen MA, Traore T, Adams-Duffy J, Thomas MP, Berger AJ, Dang L et al. MLN4924, a NEDD8-activating enzyme inhibitor, is active in diffuse large B-cell lymphoma models: rationale for treatment of NF-{kappa}B-dependent lymphoma. Blood 2010; 116: 1515–1523. 20525923 10.1182/blood-2010-03-272567

[bib14] Godbersen JC, Humphries LA, Danilova OV, Kebbekus PE, Brown JR, Eastman A et al. The Nedd8-activating enzyme inhibitor MLN4924 thwarts microenvironment-driven NF-kappaB activation and induces apoptosis in chronic lymphocytic leukemia B cells. Clin Cancer Res 2014; 20: 1576–1589. 24634471 10.1158/1078-0432.CCR-13-0987 PMC3960291

[bib15] Yao WT, Wu JF, Yu GY, Wang R, Wang K, Li LH et al. Suppression of tumor angiogenesis by targeting the protein neddylation pathway. Cell Death Dis 2014; 5: e1059. 24525735 10.1038/cddis.2014.21 PMC3944239

[bib16] Luo Z, Yu G, Lee HW, Li L, Wang L, Yang D et al. The Nedd8-activating enzyme inhibitor MLN4924 induces autophagy and apoptosis to suppress liver cancer cell growth. Cancer Res 2012; 72: 3360–3371. 22562464 10.1158/0008-5472.CAN-12-0388

[bib17] Zhao Y, Xiong X, Jia L, Sun Y. Targeting Cullin-RING ligases by MLN4924 induces autophagy via modulating the HIF1-REDD1-TSC1-mTORC1-DEPTOR axis. Cell Death Dis 2012; 3: e386. 22951983 10.1038/cddis.2012.125 PMC3461362

[bib18] Tomlins SA, Rhodes DR, Perner S, Dhanasekaran SM, Mehra R, Sun XW et al. Recurrent fusion of TMPRSS2 and ETS transcription factor genes in prostate cancer. Science 2005; 310: 644–648. 16254181 10.1126/science.1117679

[bib19] Sobel RE, Sadar MD. Cell lines used in prostate cancer research: a compendium of old and new lines—part 1. J Urol 2005; 173: 342–359. 15643172 10.1097/01.ju.0000141580.30910.57

[bib20] Sur S, Pagliarini R, Bunz F, Rago C, Diaz LA Jr, Kinzler KW et al. A panel of isogenic human cancer cells suggests a therapeutic approach for cancers with inactivated p53. Proc Natl Acad Sci USA 2009; 106: 3964–3969. 19225112 10.1073/pnas.0813333106 PMC2656188

[bib21] Brenner JC, Ateeq B, Li Y, Yocum AK, Cao Q, Asangani IA et al. Mechanistic rationale for inhibition of poly(ADP-ribose) polymerase in ETS gene fusion-positive prostate cancer. Cancer Cell 2011; 19: 664–678. 21575865 10.1016/j.ccr.2011.04.010 PMC3113473

[bib22] Lunardi A, Varmeh S, Chen M, Taulli R, Guarnerio J, Ala U et al. Suppression of CHK1 by ETS Family Members Promotes DNA Damage Response Bypass and Tumorigenesis. Cancer Discov 2015; 5: 550–563. 25653093 10.1158/2159-8290.CD-13-1050 PMC6010310

[bib23] Bridges KA, Hirai H, Buser CA, Brooks C, Liu H, Buchholz TA et al. MK-1775, a novel Wee1 kinase inhibitor, radiosensitizes p53-defective human tumor cells. Clin Cancer Res 2011; 17: 5638–5648. 21799033 10.1158/1078-0432.CCR-11-0650 PMC3167033

[bib24] Jia L, Soengas MS, Sun Y. ROC1/RBX1 E3 ubiquitin ligase silencing suppresses tumor cell growth via sequential induction of G2-M arrest, apoptosis, and senescence. Cancer Res 2009; 69: 4974–4982. 19509229 10.1158/0008-5472.CAN-08-4671 PMC2744327

[bib25] Mullenders J, von der Saal W, van Dongen MM, Reiff U, van Willigen R, Beijersbergen RL et al. Candidate biomarkers of response to an experimental cancer drug identified through a large-scale RNA interference genetic screen. Clin Cancer Res 2009; 15: 5811–5819. 19723642 10.1158/1078-0432.CCR-09-0261

[bib26] Pierce NW, Lee JE, Liu X, Sweredoski MJ, Graham RL, Larimore EA et al. Cand1 promotes assembly of new SCF complexes through dynamic exchange of F box proteins. Cell 2013; 153: 206–215. 23453757 10.1016/j.cell.2013.02.024 PMC3656483

[bib27] Tomlins SA, Mehra R, Rhodes DR, Cao X, Wang L, Dhanasekaran SM et al. Integrative molecular concept modeling of prostate cancer progression. Nat Genet 2007; 39: 41–51. 17173048 10.1038/ng1935

[bib28] Uhlén M, Björling E, Agaton C, CA-K Szigyarto, Amini B, Andersen E et al. A human protein atlas for normal and cancer tissues based on antibody proteomics. Mol Cell Proteomics 2005; 4: 1920–1932. 16127175 10.1074/mcp.M500279-MCP200

[bib29] Winston JT, Strack P, Beer-Romero P, Chu CY, Elledge SJ, Harper JW. The SCFβ-TRCP–ubiquitin ligase complex associates specifically with phosphorylated destruction motifs in IκBα and β-catenin and stimulates IκBα ubiquitination *in vitro*. Genes Dev 1999; 13: 270–283. 9990852 10.1101/gad.13.3.270 PMC316433

[bib30] Wu L, Zhao JC, Kim J, Jin HJ, Wang CY, Yu J. ERG is a critical regulator of Wnt/LEF1 signaling in prostate cancer. Cancer Res 2013; 73: 6068–6079. 23913826 10.1158/0008-5472.CAN-13-0882 PMC3790861

[bib31] Emerling BM, Weinberg F, Liu J-L, Mak TW, Chandel NS. PTEN regulates p300-dependent hypoxia-inducible factor 1 transcriptional activity through Forkhead transcription factor 3a (FOXO3a). Proc Natl Acad Sci USA 2008; 105: 2622–2627. 18268343 10.1073/pnas.0706790105 PMC2268186

[bib32] Nguyen DP, Li J, Yadav SS, Tewari AK. Recent insights into NF-kappaB signalling pathways and the link between inflammation and prostate cancer. BJU Int 2014; 114: 168–176. 24215139 10.1111/bju.12488

[bib33] Wang J, Cai Y, Shao LJ, Siddiqui J, Palanisamy N, Li R et al. Activation of NF-{kappa}B by TMPRSS2/ERG fusion isoforms through Toll-like receptor-4. Cancer Res 2011; 71: 1325–1333. 21169414 10.1158/0008-5472.CAN-10-2210 PMC3041849

[bib34] Tian F, Zhou P, Kang W, Luo L, Fan X, Yan J et al. The small-molecule inhibitor selectivity between IKK α and IKK β kinases in NF-κ B signaling pathway. J Recept Sig Transd 2015; 35: 307–318. 10.3109/10799893.2014.980950 25386663

[bib35] Bain J, Plater L, Elliott M, Shpiro N, Hastie CJ, Mclauchlan H et al. The selectivity of protein kinase inhibitors: a further update. Biochem J 2007; 408: 297–315. 17850214 10.1042/BJ20070797 PMC2267365

[bib36] Tenbaum SP, Ordonez-Moran P, Puig I, Chicote I, Arques O, Landolfi S et al. beta-catenin confers resistance to PI3K and AKT inhibitors and subverts FOXO3a to promote metastasis in colon cancer. Nat Med 2012; 18: 892–901. 22610277 10.1038/nm.2772

[bib37] Heinlein CA, Chang C. Androgen receptor in prostate cancer. Endocr Rev 2004; 25: 276–308. 15082523 10.1210/er.2002-0032

[bib38] Whitacre DC, Chauhan S, Davis T, Gordon D, Cress AE, Miesfeld RL. Androgen induction of *in vitro* prostate cell differentiation. Cell Growth Differ 2002; 13: 1–11. 11801526

[bib39] Antony L, van der Schoor F, Dalrymple SL, Isaacs JT. Androgen receptor (AR) suppresses normal human prostate epithelial cell proliferation via AR/beta-catenin/TCF-4 complex inhibition of c-MYC transcription. Prostate 2014; 74: 1118–1131. 24913829 10.1002/pros.22828 PMC4856018

[bib40] Kokontis JM, Lin HP, Jiang SS, Lin CY, Fukuchi J, Hiipakka RA et al. Androgen suppresses the proliferation of androgen receptor-positive castration-resistant prostate cancer cells via inhibition of Cdk2, CyclinA, and Skp2. PLoS ONE 2014; 9: e109170. 25271736 10.1371/journal.pone.0109170 PMC4182885

[bib41] Gan W, Dai X, Lunardi A, Li Z, Inuzuka H, Liu P et al. SPOP promotes ubiquitination and degradation of the ERG oncoprotein to suppress prostate cancer progression. Mol Cell 2015; 59: 917–930. 26344095 10.1016/j.molcel.2015.07.026 PMC4575912

[bib42] An J, Ren S, Murphy SJ, Dalangood S, Chang C, Pang X et al. Truncated ERG oncoproteins from TMPRSS2-ERG fusions are resistant to SPOP-mediated proteasome degradation. Mol Cell 2015; 59: 904–916. 26344096 10.1016/j.molcel.2015.07.025

[bib43] Wang J, Cai Y, Yu W, Ren C, Spencer DM, Ittmann M. Pleiotropic biological activities of alternatively spliced TMPRSS2/ERG fusion gene transcripts. Cancer Res 2008; 68: 8516–8524. 18922926 10.1158/0008-5472.CAN-08-1147 PMC2597580

[bib44] An J, Wang C, Deng Y, Yu L, Huang H. Destruction of full-length androgen receptor by wild-type SPOP, but not prostate-cancer-associated mutants. Cell Rep 2014; 6: 657–669. 24508459 10.1016/j.celrep.2014.01.013 PMC4361392

[bib45] Geng C, Rajapakshe K, Shah SS, Shou J, Eedunuri VK, Foley C et al. Androgen receptor is the key transcriptional mediator of the tumor suppressor SPOP in prostate cancer. Cancer Res 2014; 74: 5631–5643. 25274033 10.1158/0008-5472.CAN-14-0476 PMC4209379

[bib46] Yu J, Yu J, Mani RS, Cao Q, Brenner CJ, Cao X et al. An integrated network of androgen receptor, polycomb, and TMPRSS2-ERG gene fusions in prostate cancer progression. Cancer Cell 2010; 17: 443–454. 20478527 10.1016/j.ccr.2010.03.018 PMC2874722

[bib47] Sun C, Dobi A, Mohamed A, Li H, Thangapazham RL, Furusato B et al. TMPRSS2-ERG fusion, a common genomic alteration in prostate cancer activates C-MYC and abrogates prostate epithelial differentiation. Oncogene 2008; 27: 5348–5353. 18542058 10.1038/onc.2008.183 PMC7556723

[bib48] Morris MJ, Huang D, Kelly WK, Slovin SF, Stephenson RD, Eicher C et al. Phase 1 trial of high-dose exogenous testosterone in patients with castration-resistant metastatic prostate cancer. Eur Urol 2009; 56: 237–244. 19375217 10.1016/j.eururo.2009.03.073 PMC2738932

[bib49] Lee YG, Korenchuk S, Lehr J, Whitney S, Vessela R, Pienta KJ. Establishment and characterization of a new human prostatic cancer cell line: DuCaP. *in vivo* 2001; 15: 157–162. 11317521

[bib50] Tan SH, Furusato B, Fang X, He F, Mohamed AA, Griner NB et al. Evaluation of ERG responsive proteome in prostate cancer. Prostate 2014; 74: 70–89. 24115221 10.1002/pros.22731 PMC4075339

