# Supplementary material for: Distinct outcomes of CRL–Nedd8 pathway inhibition reveal cancer cell plasticity
Source: Cell Death Dis. 2016 Dec 1;7(12):e2505–. doi: 10.1038/cddis.2016.395 (PMC5261022; doi:10.1038/cddis.2016.395)
Supplement: Supplementary Information [file cddis2016395x1.pdf]

SUPPLEMENTARY INFORMATION

Distinct outcomes of CRL-Nedd8 pathway inhibition reveal cancer cell plasticity

*Anastasia V. Rulina[1,2,3], Frédérique Mittler[1,2,3], Patricia Obeid[1,2,3], Sophie Gerbaud[1,2,3], Laurent Guyon[1,2,3], Eric Sulpice[1,2,3], Frédérique Kermarrec[1,2,3], Nicole Assard[1,2,3], Monika E. Dolega[1,2,3], Xavier Gidrol[1,2,3], and Maxim Y.Balakirev[1,2,3]**

[1]Commissariat a l'Energie Atomique et aux Energies Alternatives (CEA), iRTSV-BGE-BIOMICS, F-38000 Grenoble, France.
[2]University Grenoble Alpes, iRTSV-BGE, F-38000 Grenoble, France.
[3]INSERM, BGE, F-38000 Grenoble, France.
*Corresponding author, maxim.balakirev@cea.fr

RUNNING TITLE: **CRL inhibition in prostate cancer cells**

TABLE OF CONTENTS

	Pages
**Supplementary Methods**..	2-7
**Supplementary Figures S1-S14**..	8-21
**Supplementary Tables S1-S5**..	22-28
**Supplementary References**..	29

CONCLUSION

This paper demonstrates that the inhibition of neddylation is a fairly non-specific pan-CRL approach that may promote three very different outcomes: be specifically toxic to cancer cells; non-specific; and even potentially dangerous by promoting more aggressive phenotypes. The latter conclusion comes from our observation that MLN stimulates the pro-metastatic Wnt/β-Cat–FoxO pathway. Nevertheless, the knowledge of the underlying molecular mechanisms will help to design complementary regimens that may reliably shift the balance in prostate cancer treatment toward positive patient outcomes.

SUPPLEMENTARY METHODS

Cell culture

The LNCaP (AR+, androgen-dependent, p53-wt, PTEN-mut), PC3 (AR-, androgen-independent, p53-null, PTEN-null), and VCaP (both AR+, androgen-sensitive, contain TER mutation, p53-R248W, PTEN-wt) cell lines were purchased from the American Type Culture Collection (ATCC). The DuCaP cell line (similar origin as VCaP) was kindly provided by Prof. Jack Schalken from the Radboud University Nijmegen Medical Center, who originally received them from Kenneth J. Pienta, MD, Director of Research at The Brady Urological Institute, Baltimore, where this cell line was created (Lee et al, 2001). Cell lines were used within 6 months after receipt and were tested on a regular basis for AR, PSA, SLC45A3, and TMPRSS-ERG by immunoblotting and quantitative RT-PCR. VCaP and DuCaP cells were cultured in DMEM (Gibco, 41966) containing 10% FBS (PAN Biotech, P30-3302) and 1% penicillin/streptomycin (Gibco, 15140). PC3 and LNCaP cells were cultured in RPMI1640 (Gibco, 61870) with the same supplements. The cells were then grown in the incubator at 37°C with 5% CO_2. For different passages, the cells were washed twice with PBS (no calcium, no magnesium, Gibco, 14190) followed by the addition of trypsin-EDTA (Gibco, 25300) and incubation for 3-10 minutes depending on cell line. Subculture was done depending on the density of the cells. Usually for VCaP cells, subculture was done once per week, with dilution to 1/2; DuCaP was once per week, with dilution to 1/10; LNCaP, PC3 and RWPE1 were twice per week, with dilution to 1/5.

Charcoal stripped serum

Charcoal/dextran stripping removes non-polar material such as lipophilic materials (virus, certain growth factors, hormones and cytokines) but has little effect on salts, glucose, amino acids, etc. Dextran coated charcoal was prepared by stirring 2.5% (w/v) Norit-A charcoal and dextran T-70 (0.25% w/v) into PBS and incubating for 18 hours at 4°C. The dextran-coated charcoal was pelleted by centrifugation at 1,000 g for 5 minutes. The supernatant was drained off and replaced with the same volume of Fetal Bovine Serum. The mixture was vortexed, to thoroughly mix the charcoal with the serum, and then incubated for 12 hours at 4°C. The resulting mixture was passed through a prefilter and 0.45 micron filter before sterilizing through a 0.2 micron filter. The stripped serum was aliquoted by 50 ml and stored at -20°C.

Spheroid culture

Cells were suspended in the standard medium, and distributed into ultra-low attachment U-bottom plates (Falcon, 353910) at concentration of 500 cells/ 100 µl per well. This resulted in formation of spheroids in 2 days with an average diameter of 200 µm. Image acquisitions were made with a change of culture medium once a week. Prior to the addition of fresh medium, the old culture medium was removed using a multichannel pipet.

Cellular ATP assay

Cell metabolism was analyzed by measuring ATP content using ViaLight™ Plus Cell Proliferation and Cytotoxicity BioAssay Kit from Lonza (LT07-121) according to the manufacturer's protocol. In short, cells were seeded in white plates with transparent bottom suitable for luminescence assays (Grenier,

655088). The treatments were performed on the next day after cell seeding. At indicated times, the lysis reagent was added to the cells directly into culture medium for 10 minutes, followed by addition of ATP monitoring reagent for 5 minutes. Luminescence was measured using GloMax®-Multi Detection System (Promega).

Apoptosis assay

The increase in the number of apoptotic cells was estimated using CellEvent™ Caspase-3/7 Green Detection Reagent from Invitrogen (C10423). This involved the cells being seeded in black plates with transparent bottoms suitable for fluorescent measurements (Fisher Scientific, 781091). The treatments were performed on the day after cell seeding. CellEvent reagent was added during the treatment according to the manufacturer's protocol. At the end of treatment, Hoechst dye was added, and cells were incubated for 30 minutes. The image acquisitions were performed using CellInsight™ NXT High Content Screening Platform (Thermo Scientific). The images were analyzed and quantified by "Cell Health Profiling" program, installed within CellInsight™. Segmentation is based on the detection of nuclei with the Hoechst channel. Using this segmentation, the CellEvent signal was then quantified in each nucleus. The apoptotic cells were determined by the increase of the signal from CellEvent reagent (see below, "Data quantification").

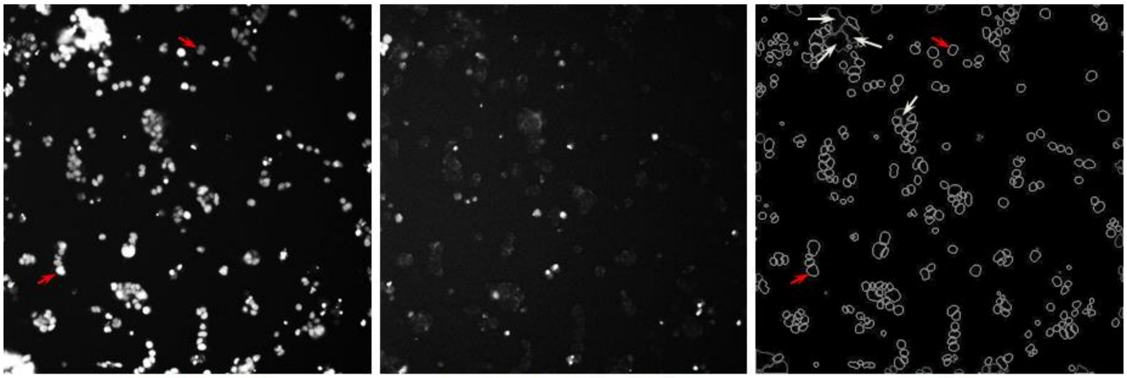

Nuclear segmentation in CellEvent-based apoptosis assay with CellInsight. A, Nuclei staining with Hoechst dye. B, Fluorogenic CellEvent reagent is accumulated in the nucleus upon cleavage by caspases 3/7. C, Segmentation using "Cell Health Profiling" software. In ambiguous cases, software excludes some areas from the analysis (white arrows), while keeping the others (red arrows).

Data quantification

The effect of each treatment was measured in 4 well replicates split into two 384 (or 96)-microwell plates (2 replicates per plate). The fluorescent signals (Hoechst and CellEvent) were measured in 9 fields per well-replicate. Based on these signals, the "Cell Health Profiling" program performs cell segmentation and obtains information about each individual cell, including the intensity of the signal from the channel (Hoechst and CellEvent), the area of the nuclei and the total number of cells per field. Based on the CellEvent signal intensities from negative siAllStars and positive siCellDeath controls, the threshold that distinguishes live cells from apoptotic was established. Based on this threshold, the median value of the percentage of dying cells was then calculated.

Statistical analyses have been performed using the statistical software R (http://www.r-project.org/). Box and whiskers plots represent the distribution of the data, with the box delimiting the central half of the data (from first to third quartiles); the segment is the median of the data. The whiskers delimit the rest of the data if its length does not exceed 1.5 times the size of the box, other data points are indicated by circles. P-values are calculated using the two-sided Wilcoxon rank-based test.
For analysis and comparison of the effects of siRNA in various cell lines we used robust Z-score (RZ), which allows the data to be less dependent on "outliers". The Z-score is the distance from the mean of the whole plate normalized by its standard deviation. Its robust version, RZ, calculates as follow (Birmingham et al, 2009):

$$RZ_w = \frac{X_w - Md}{MAD*k}$$

where $\mathbf{RZ_w}$ is a robust Z-score for the well w, $\mathbf{X_w}$ is the value for the well (either percentage of dying cells or cell number); **Md** is a median value of the X_w for the whole plate, **MAD** is a median absolute deviation of X_w for the whole plate and **k** is the constant scale factor, which depends on the distribution, and is equal to 1.4826 in case of the Gaussian distribution.

DNA synthesis assay by EdU incorporation

The quantity of proliferating cells in the population was determined by measurement of DNA synthesis (by EdU incorporation) using Click-iT® EdU Alexa Fluor® 647 Flow Cytometry Assay Kit from Invitrogen (C-10419). This involved the cells being seeded in black plates with transparent bottoms suitable for fluorescent measurements (Fisher Scientific, 781091). The treatments were performed on the day after cell seeding. At indicated times, VCaP cells were treated with EdU for 5 hours and then fixed and stained according to the manufacturer's protocol. Finally, Hoechst reagent was added to the cells followed by incubation for 30 minutes. The labeled cells were covered with Glycerol and PBS++ solution (in a ratio 1:1) and stored at 4°C. Image acquisitions were performed using CellInsight™ NXT High Content Screening Platform (Thermo Scientific). The images were analyzed and quantified by the "Cell Health Profiling" program, installed within CellInsight™. The identification of cells (cell segmentation) was based on the detection of nuclei by the Hoechst channel. The EdU signal was quantified for each nucleus, and the results were presented as a percentage of cells having nuclear EdU staining above a threshold.

Cell-cycle analysis

The cell cycle was analyzed by the measurement of total DNA content using flow cytometry. Cells were grown in culture medium with or without drug treatment. They were harvested with trypsin, neutralized by culture medium and washed once in PBS. Then the cells were fixed with 70% fridge-cold ethanol for 30 minutes, and BSA was added to a final concentration of 0.5%. The cells were spun at 3000 rpm for 7 minutes, the supernatant was discarded, and the cells were resuspended in 0.25 % BSA in PBS and spun again at 3000 rpm 7 minutes. The supernatant was discarded, and replaced by a 50 µg/ml 7-AAD (7-aminoactinomycin D) solution in PBS. The 7-AAD labeled cells were analyzed by BD™ LSR II flow cytometer from BD Biosciences.

Senescence test

Analysis of senescence was performed by measuring of β-galactosidase activity according to the previously described protocol (Debacq-Chainiaux et al, 2009). The protocol was slightly modified when the test was performed with spheroids. This involved the suspension of VCaP cells being distributed in ultra-low attachment U-bottom plates (Falcon, 353910) in concentrations of 500 cells/well/100 µl and being incubated during 10 days. Typically, 30 spheroids per condition were used. Next, the spheroids were harvested, pelleted at 600 rpm for 5 min, resuspended in 150 µl of 1.5% low-melting agarose (Sigma, A9414) and distributed into Lab Tek chambers (Dominique Dutscher, 055082). After polymerization for about 30 minutes, the gels were washed twice with PBS, fixed with 2% formaldehyde and 0.2% glutaraldehyde in PBS during 7 minutes. This was followed by a double wash with PBS, and the addition of a staining solution (containing citric acid/Na phosphate buffer, 5 mM $K_4[Fe(CN)_6]x3H_2O$, 5 mM $K_3[Fe(CN)_6]$, 150 mM sodium chloride, 2 mM magnesium chloride and 1 mg/ml X-gal in distilled water). The spheroids were then incubated at 37°C during 5 hours. Then the gels were washed multiple times with PBS to remove the background yellow staining of agarose. As a final step, the gels were washed with methanol for 1 min and viewed by bright field microscopy.

Immunofluorescence microscopy

Cells were grown on plasma-treated glass slides. The culture medium was removed and replaced by 4% paraformaldehyde (PFA) for 15 min at RT. Then PFA was replaced with 0.2% Tween for 5 min at RT. Treatment with Tween was followed by the addition of NH_4Cl 0,1M for 10 min at RT. Then the slides were washed briefly in PBS +Ca^{2+} +Mg^{2+} (PBS++, Sigma, P4417) and blocked with 3 % BSA in PBS++ which had been filtered through a 0.2 µm filter for 30 min. Primary antibodies were added in 1.5 % BSA (filtered) and left for 2 hours at RT. This was followed by 3 washes with PBS++, and then the slides were incubated with secondary antibodies and phalloidin in 1.5 % BSA (filtered) for 45 min at RT. This was followed by a 5 min wash in PBS++, then a 5 min wash in Hoechst, and then again 5 min with PBS++. Then, these glass slides were placed on standard microscope slides with mounting solution (DAKO, S302380) and dried for 24 hours. Finally, the slides were analyzed by Zeiss Axioimager Z1 Apotome from Zeiss.

Western blotting and ELISA

Cellular proteins were extracted using RIPA lysis buffer (Sigma, R0278) complemented with protease inhibitor cocktail (Complete Mini from Roche Diagnostics, Cat. No. 11 836 153 001) and additional inhibitors (10 mM *ortho*-phenanthroline, 30 mM N-Ethylmaleimide, 5 mM sodium ortho-vanadate, and 5 mM sodium fluoride). After quantification with a BCA protein assay kit (Pierce, 23225), an equal range of concentration (typically 2.5 ng of protein per sample) was run on a NuPAGE Novex Bis-Tris Gel (Life Technologies, NP0322BOX, EC60252BOX, NP0323BOX) in MES buffer and then transferred onto the nitrocellulose membrane (Amersham™ Protran®, GE Healthcare, 10600001). The membranes were blocked in 5% nonfat milk/TBST for 40 min at 37°C, incubated with primary antibodies in 5% nonfat milk/TBST for 1 hour at RT or overnight at 4°C. This step was followed by incubation with secondary HRP-conjugated antibodies. Detection was performed with a chemiluminescent reagent depending on the concentration of the target protein (Plus-ECL, Perkin Elmer, NEL105001EA; ECL Prime, GE Healthcare, RPN2232; SuperSignal West Femto Substrate, Thermo Fisher Scientific, 34095). Secreted

PSA was analyzed with Anogen Human Free PSA ELISA Kit. A list of antibodies is given in **Supplementary Table S1**.

siRNA transfection

Cells were transfected with siRNA using Lipofectamine® RNAiMAX Transfection Reagent (Invitrogen, 13778) according to the manufacturer's protocol with minor modifications: RNAiMAX was taken 0.75 µl per well of 96-well plate and 386-well plate. Screening of the CRL genes was performed using siRNAs from ON-TARGETplus® SMART pool® siRNA Library-Human Ubiquitin Conjugation Subset 1, complemented with RBX1 and SAG(RBX2) ON-TARGETplus® SMART pool® siRNAs from Dharmacon. Transfection of the SMART pool was done at a final concentration of 20 nM of siRNA and individual siRNAs were added in concentrations of 10 nM, unless otherwise indicated. The optimal knockdown effect was observed with 3-day (LNCaP, PC3) or 5-day (VCaP) siRNA treatment. As controls for transfection AllStars Negative Control siRNA (SI03650318, Qiagen) and AllStars Hs Cell Death siRNA Positive cell death phenotype control (SI04381048, Qiagen) were used. All controls were used in concentrations equal to the concentration of siRNA in the experiment. The siRNA against the ERG gene was prepared by Eurogentec. The sequences of siERG were taken from publication of Tan *et al.*, 2009 (Tan et al, 2014): sense - 5'-CGACAUCCUUCUCUCACAUAU-3'; antisense - 5'-AUGUGAGAGAAGGAUGUCGUG -3'. Three siRNAs for AR were obtained from Dharmacon and used as a pool. siRNA sequences are listed in **Supplementary Table S2**.

Luciferase Reporter Assays

Cells were grown in white 96-well plates with transparent bottom suitable for luminescence assays (Grenier, 655088) until they reached 70%-80% confluence. Next, cells were co-transfected in triplicates with a transcription factor-specific Firefly luciferase reporter and a constitutively-active Renilla luciferase reference vector (1:10 w/w ratio, 200 ng of total DNA per well) and with 0.5 µl/ well Lipofectamine® 2000 Transfection Reagent (ThemoFisher, 11668019) according to the manufacturer's protocol. The cells were treated with MLN on the day after transfection and analyzed after 24 hours. Luciferase measurements were performed using the Dual-Luciferase Reporter Assay (Promega), according to the manufacturer's instructions using GloMax®-Multi Detection System (Promega). The ratio of Firefly- to Renilla-luciferase activities was calculated. All values were presented as means ± SD. Reporter plasmids are listed in **Supplementary Table S3**.

RNA extraction, RT-PCR, and qPCR

RNA was extracted with an RNeasy Mini Kit (QIAGEN, 74104). 1.5 µg RNA was reverse-transcribed in a total volume of 20 µl using a SuperScript® VILO cDNA Synthesis Kit (Life Technologies, 11754050) with random primers according to the manufacturer's protocol. Reverse transcription reactions were diluted to 200 µl of distilled water and further used in concentrations of 2.5 µl per reaction of quantitative PCR (qPCR). qPCR was carried out with a Platinum Quantitative PCR SuperMIX-UDG Kit (Life Technologies, 11730-017) using a StepOnePlus Real-Time PCR system (Applied Biosystems, 4376600). All experiments were run in triplicates, and the results were normalized to 18S rRNA expression. Primer sequences are listed in **Supplementary Table S4**.

Chemical inhibitor screening

The effect of specific chemical inhibitors on cell viability was analyzed by measuring the number of apoptotic cells as described in "Measurement of apoptosis" section. The treatments were performed in 100 µl triplicates at ~60% of cell confluence. The inhibitors and MLN were diluted in cell medium from DMSO stocks (0.5% DMSO in final solution). Pure 0.5% DMSO was used as a control condition. CellEvent reagent was added during the treatment according to the manufacturer's protocol. At the end of treatment, Hoechst dye was added, and cells were incubated for 30 minutes. The data acquisition and analysis were performed as described above in "Measurement of apoptosis" and "Data quantification" sections. A list of inhibitors is given in **Supplementary Table S5**.

SUPPLEMENTARY FIGURES

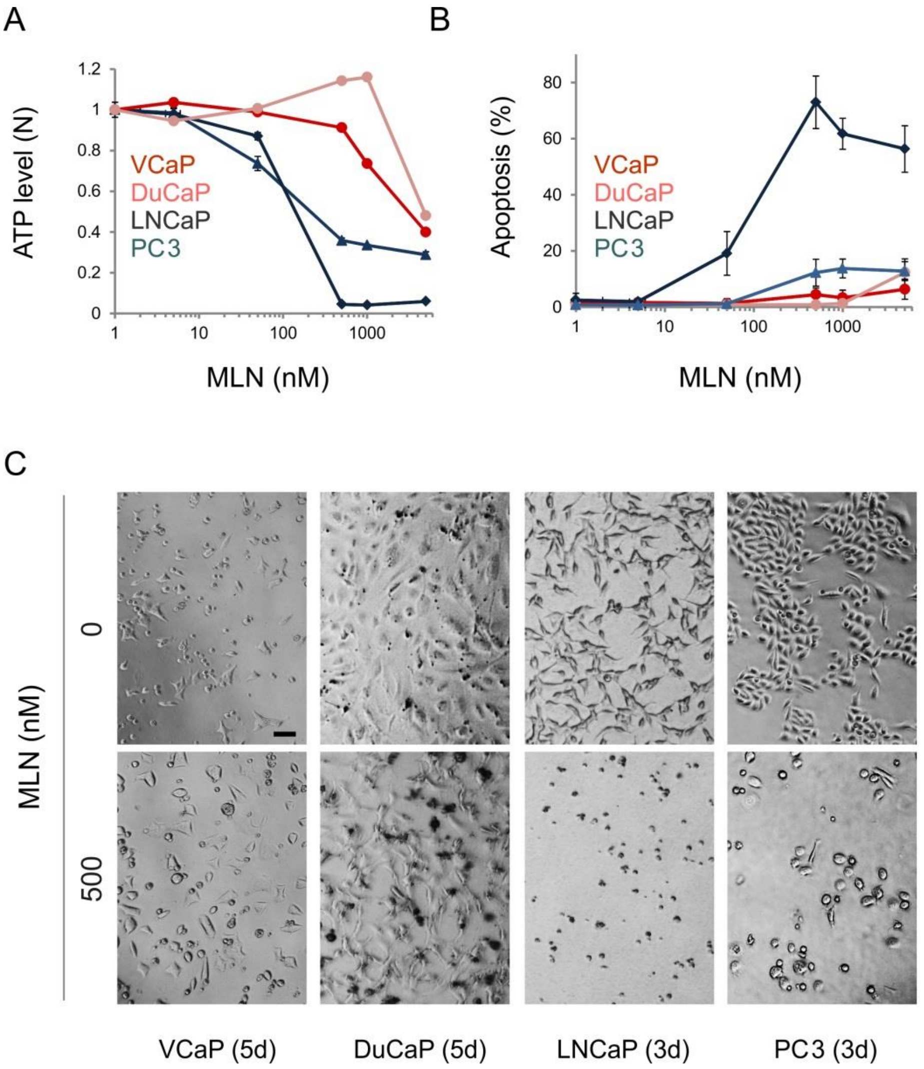

Supplementary Figure S1. Different sensitivity of prostate cancer cell lines toward MLN. A, Effect of MLN on cellular ATP content. Indicated cell lines were grown in 10% StdM for 3 days in the presence of various concentrations of MLN. Cellular ATP content was measured with ViaLight™ Plus Cell Proliferation chemiluminescent kit. The data were normalized to ATP signal measured with vehicle (DMSO)-treated cells (mean ± s.d.). B, Apoptosis induction by MLN. Indicated cell lines were grown in 10% StdM for 3 days in the presence of various concentrations of MLN. Caspase activation was measured with CellEvent™ Caspase-3/7 fluorogenic substrate (CE, green). Before measurement cells were stained with Hoechst dye (DNA, blue) and analyzed by automated fluorescence microscopy as described in Materials and Methods. The data are presented as a percentage of apoptotic cells (mean ± s.d.). C, Phase contrast microscopy images show the changes in cells morphology induced by MLN (scale bar is 100 μm).

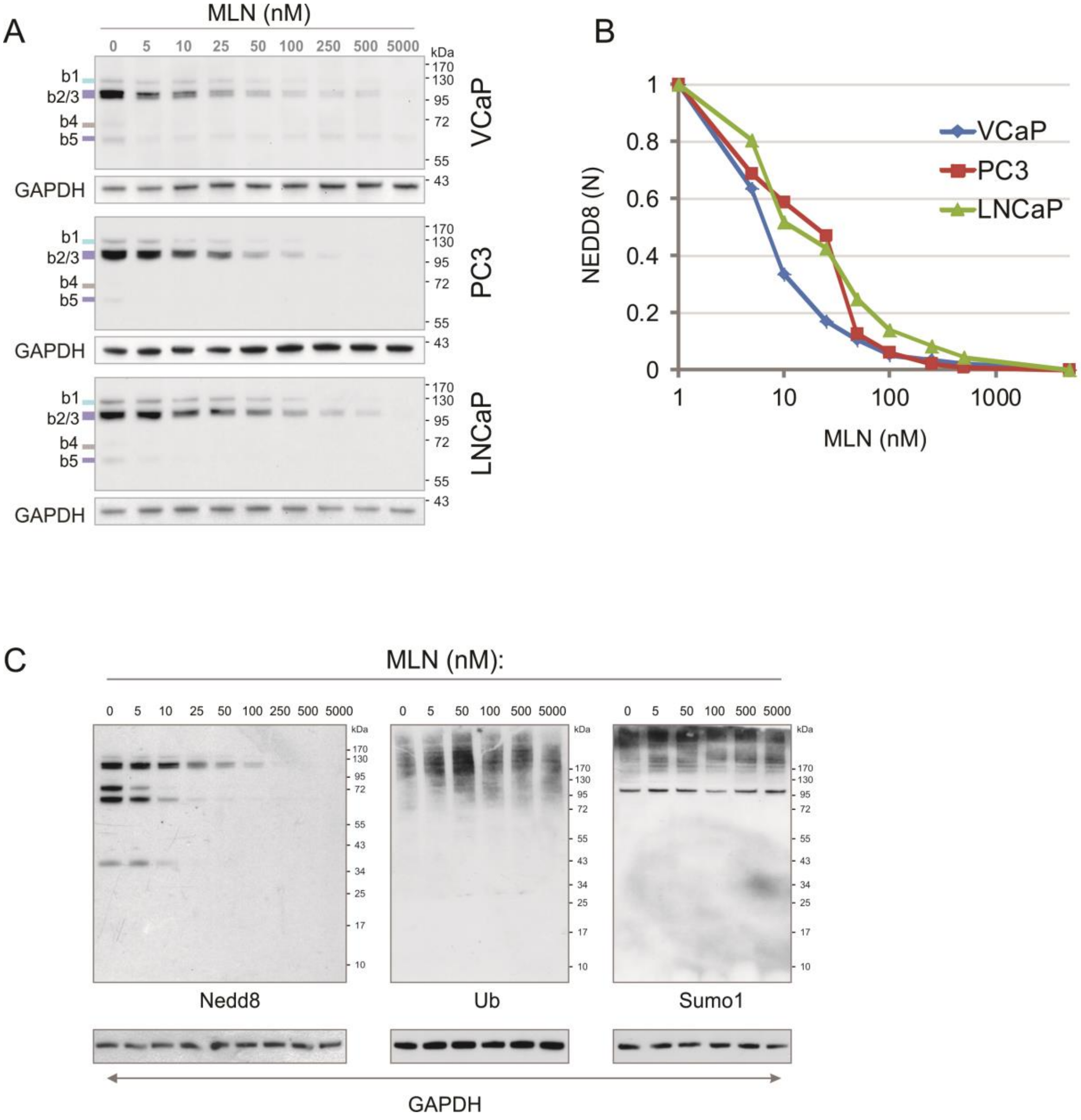

Supplementary Figure S2. Inhibition of cellular neddylation by MLN. A, Analysis of Nedd8 conjugates in three prostate cell lines. Cells were grown in10% StdM, treated with increasing concentrations of MLN for 1 d and analyzed by western blotting with Nedd8-specific antibody and anti-GAPDH for loading control. B, The abundance of neddylated proteins was quantified by ImageJ using Supplementary Figure S2A, and normalized first to GAPDH, then to vehicle control. C, The effect of MLN is Nedd8-specific. Western blot analysis of Nedd8, Ub, and Sumo1 conjugates in VCaP cells. Cells were treated with increasing concentrations of MLN and analyzed by western blotting with protein-specific antibodies and anti-GAPDH for loading control.

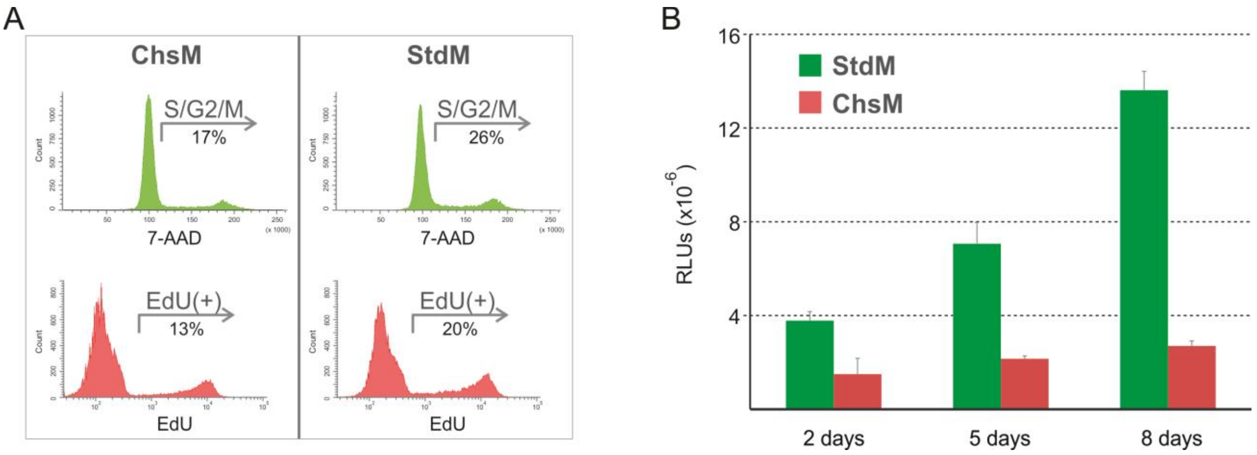

Supplementary Figure S3. Effect of charcoal-stripping of serum on the proliferation of VCaP cells. A, VCaP cells grown on 10% ChsM or StdM were labeled with nucleic acid dye 7-AAD (to measure cellular DNA content) or pulse labeled for 6h with EdU (to measure cellular DNA synthesis) and analyzed by FACS. The percentage of proliferating cells is shown on the diagram. B, Analysis of VCaP cells proliferation by measuring cellular ATP content with ViaLight™ kit. The same number of cells (15000) were inoculated in 10% ChsM or StdM and grown for indicated time.

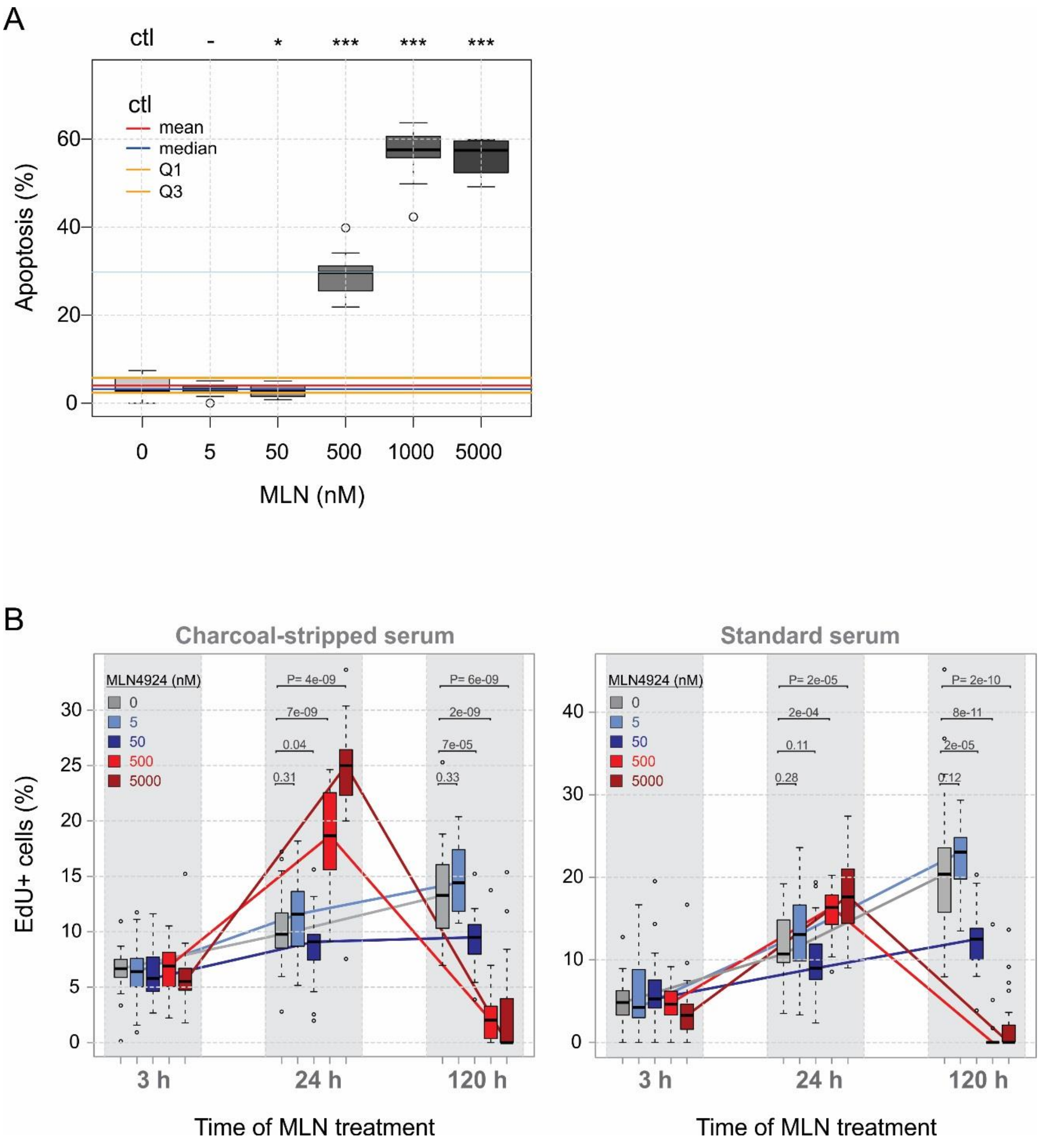

Supplementary Figure S4. Effect of MLN on cell survival and DNA synthesis. A, VCaP cells lines were grown in 10% ChSM for 5 days in the presence of various concentrations of MLN. The percentage of apoptotic cells was measured as described in Materials and Methods. The data are shown as a boxplot diagram with P-values compared to control (ctl) condition (* means $P < 0.05$, *** means $P < 0.001$, Wilcoxon test). B, Cells grown in 10% ChSM (left diagram) or 10% StdM (right diagram) were treated with MLN for indicated times and DNA synthesis was measured by EdU incorporation using Click-iT EdU technology and quantitative automated fluorescence microscopy. The percentage of EdU-positive cells is shown as a boxplot diagram along with the corresponding P-values (Wilcoxon test, see Materials and Methods for more details).

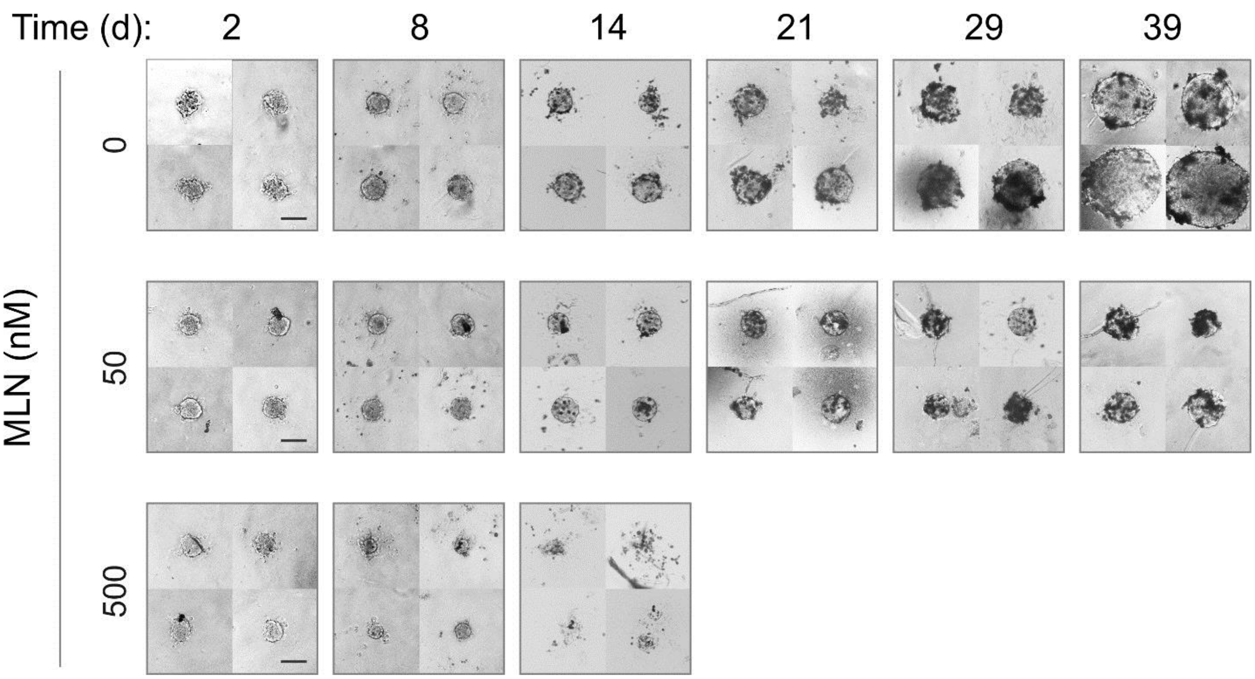

Supplementary Figure S5. Effect of MLN on spheroids growth. Phase-contract microscopy with 10x objective. Scale bar is 200 μm.

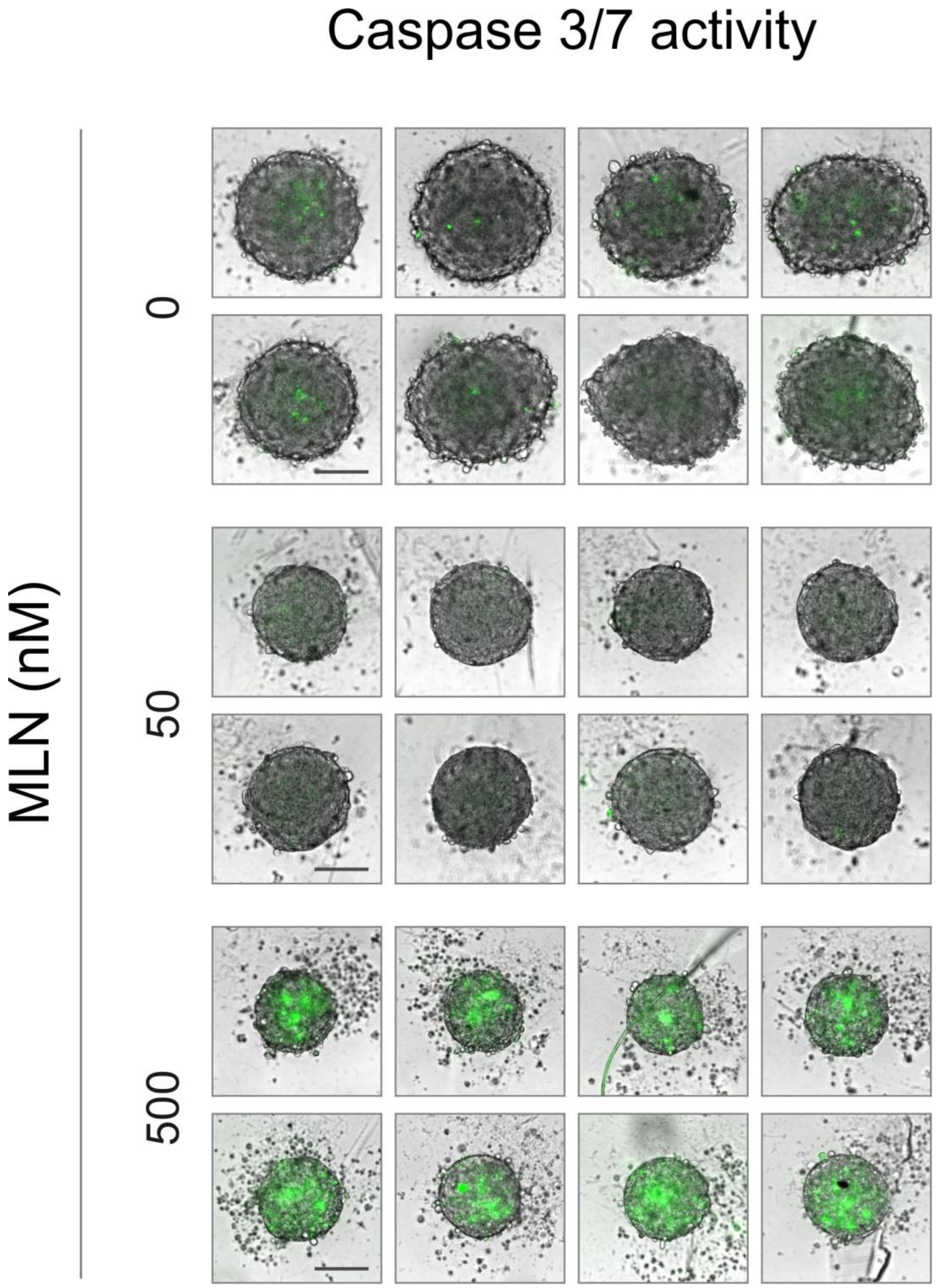

Supplementary Figure S6. MLN induces apoptosis in VCaP spheroids. Apoptosis was measured with CellEvent™ Caspase-3/7 fluorogenic substrate (cleaved substrate is seen in green on the merged phase-contract/ fluorescent microscopy images). Scale bar is 100 μm.

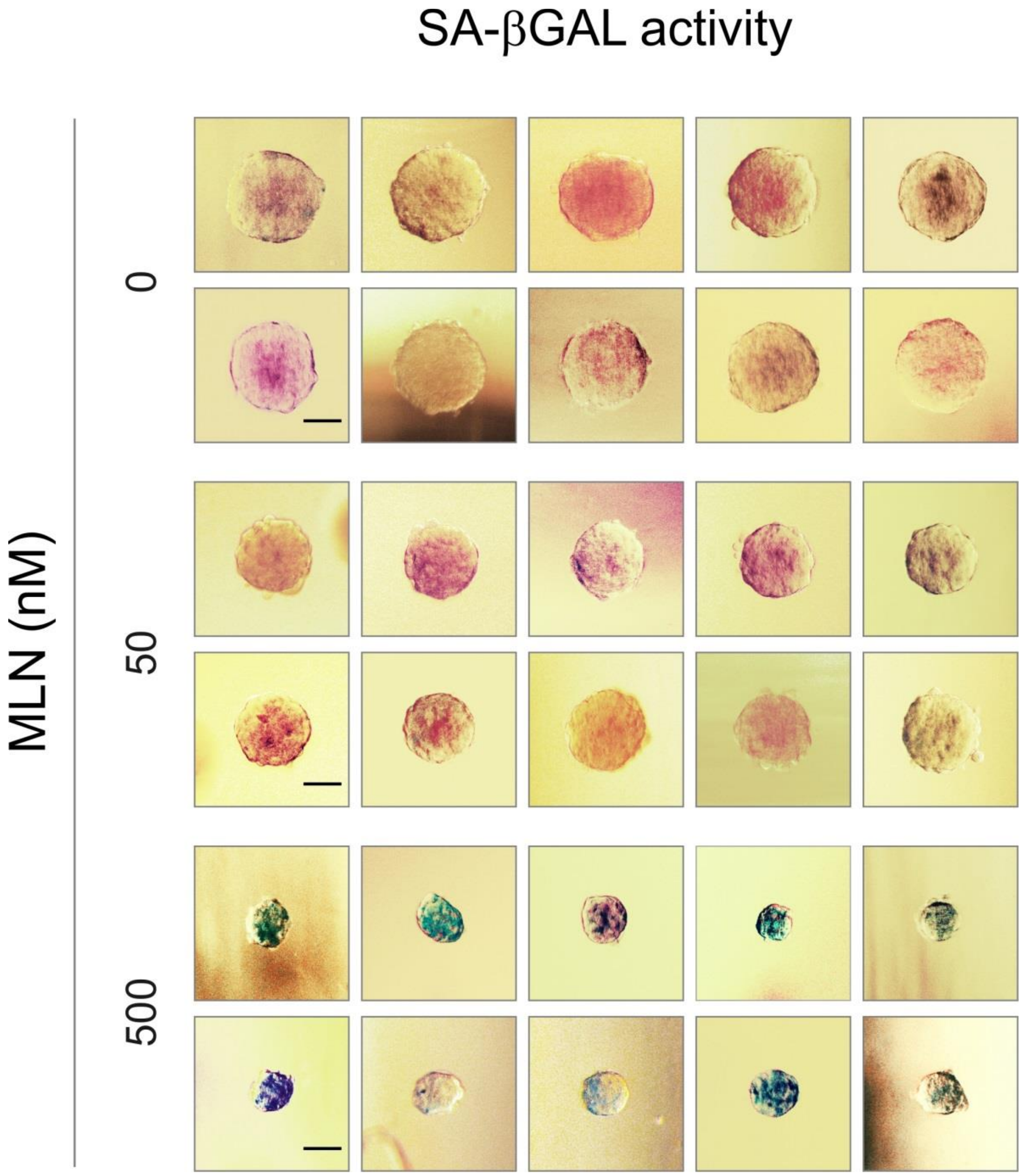

Supplementary Figure S7. MLN induces senescence in VCaP spheroids. Senescence was measured with colorimetric X-GAL substrate for senescence-associated beta-galactosidase (SA-β-GAL, cleaved substrate produced blue color). Scale bar is 100 μm.

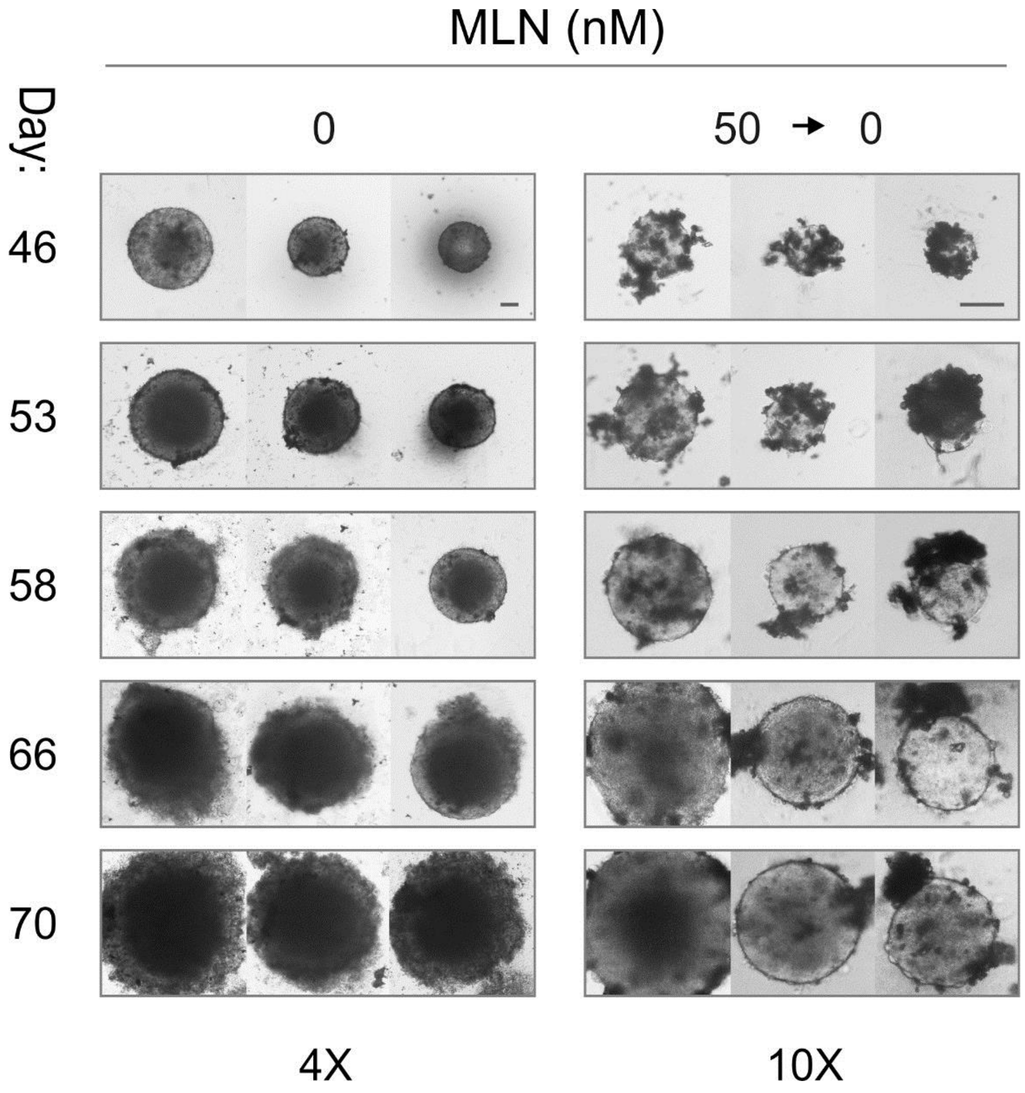

Supplementary Figure S8. Effect of MLN on spheroids growth. Phase-contract microscopy (10x objective, after 46 days 4x magnification was used for spheroids grown in MLN-free medium). After 40 days, the spheroids grown with 50 nM MLN were transferred into MLN free medium. Scale bar is 200 μm.

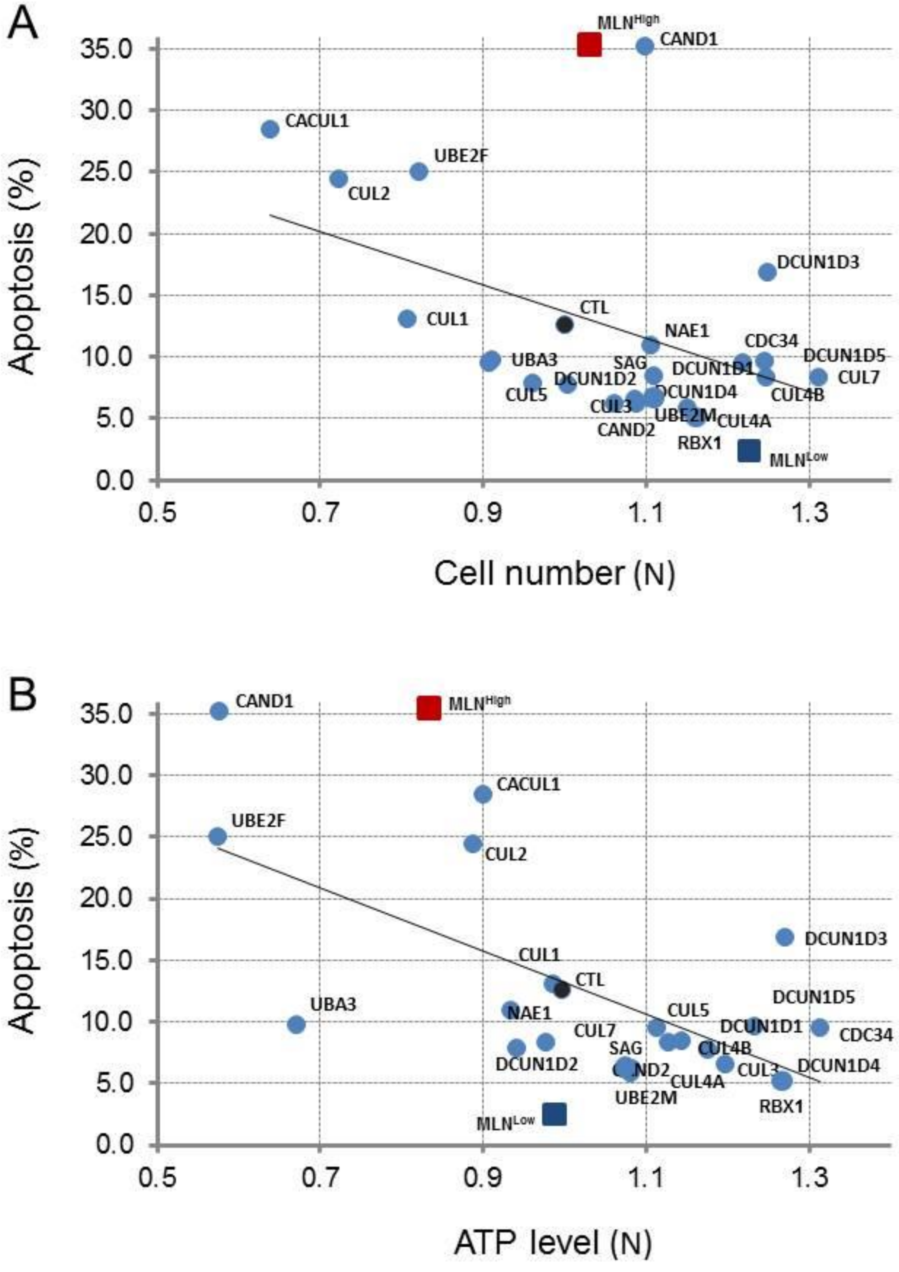

Supplementary Figure S9. Distinct roles of the components of CRL-Nedd8 pathway in cell regulation. A, Effect of gene knockdown on VCaP cell proliferation and survival. 22 genes were individually silenced using ON-TARGETplus® SMART pool® siRNAs (Dharmacon). The effect of gene knockdown was measured after 5 days of siRNA treatment and compared with that of control siRNA (siCTL, AllStars Negative Control siRNA, Qiagen) and 50 nM and 500 nM doses of MLN (MLN^{low} and MLN^{high}). The number of cells and the percentage of apoptotic cells were measured simultaneously as described in Materials and Methods. B, Cellular ATP content was measured in separate experiments as described in Materials and Methods. Cell number and ATP level were normalized to the values obtained with siCTL-treated cells.

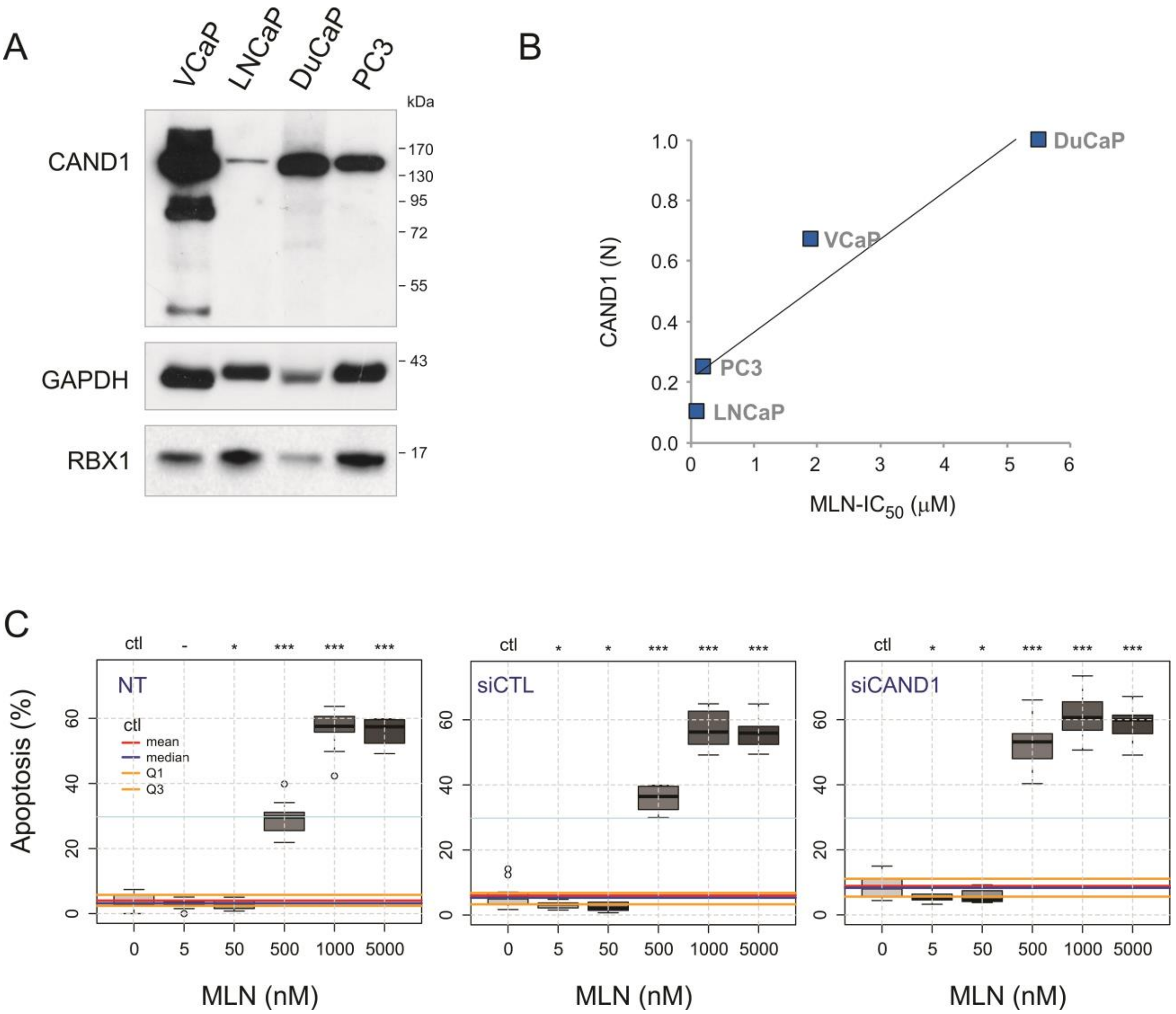

Supplementary Figure S10. MLN cytotoxicity and CAND1. A, Western blot analysis of Cand1 and RBX1 expression in four prostatic cell lines. Cell lysates were analyzed by western blotting with protein-specific antibodies and anti-GAPDH for loading control. B, Correlation between cellular level of Cand1 protein (quantified by ImageJ using Supplementary Figure S10A, and normalized to GAPDH) and the sensitivity of a cell line to MLN (IC50 values were estimated using the data shown in Figure 1A). C, CAND1 knockdown potentiates the toxic effect of MLN. VCaP cells were transfected with 5 nM of ON-TARGETplus® SMART pool® CAND1 siRNA or with the same amount of siCTL and treated with MLN on the next day. The percentage of apoptotic cells was measured as described in Materials and Methods. The data are shown as a boxplot diagram with P-values compared to control (ctl) condition (* means $P < 0.05$, *** means $P < 0.001$, Wilcoxon test). NT- non-transfected control.

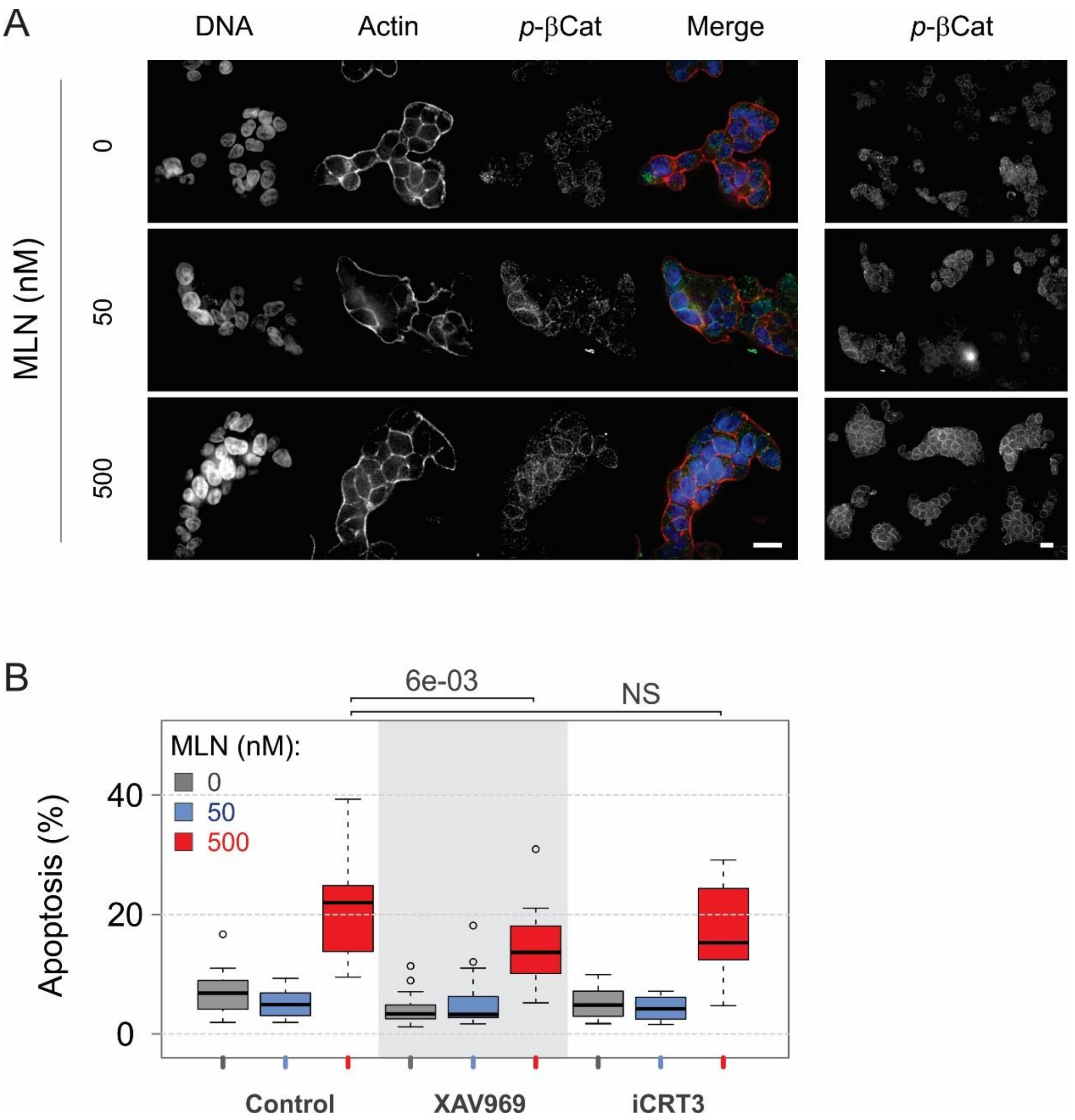

Supplementary Figure S11. MLN interaction with Wnt/β-Cat pathway. A, Immunofluorescence analysis of *p*-β-Cat (phospho-Ser33/37/Thr41) in VCaP cells treated with indicated concentrations of MLN. Cells were probed with *p*-β-Cat -specific antibody (*p*-β-Cat, green), actin-specific antibody (Actin, red), and Hoechst dye (DNA, blue). Scale bar is 20 µm. B, Effect of Wnt/β-Cat pathway inhibitors XAV969 (1 µM) and iCRT (5 µM) on MLN-induced apoptosis.

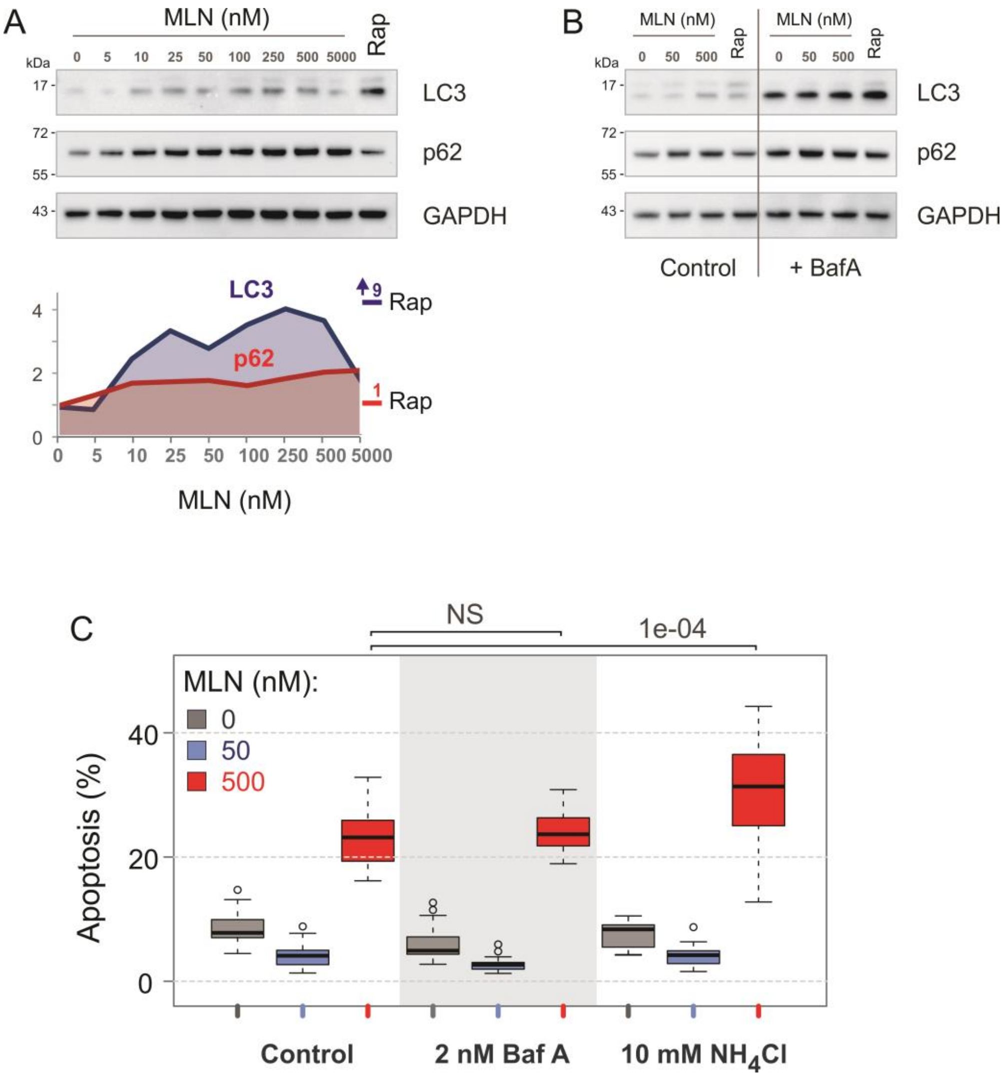

Supplementary Figure S12. MLN interaction with autophagy. A, Western blot analysis of autophagy markers LC3 and p62/SQSTM1 in VCaP cells. Cells were treated for 24 h with increasing concentrations of MLN or 100 nM of Rapamycin (Rap, autophagy stimulation control) and analyzed by western blotting on PVDF membrane with protein-specific antibodies and anti-GAPDH for loading control. The plot below shows the relative protein levels normalized first to GAPDH, then to vehicle control (the corresponding values for Rapamycin are shown on the right). B, Western blot analysis of autophagic flux. Cells were first treated with indicated concentrations of MLN or 100 nM of Rapamycin for 24 h followed by the addition of 10 nM Bafilomyicin A1 for 2 h (Baf A, an inhibitor of lysosomal H+-ATPase and degradation pathway). The control cells were not treated with Bafilomyicin A1. Cell lysates were analyzed by western blotting as above. C, Effect of autophagy inhibitors on MLN-induced apoptosis. Cells were grown in 10% ChsM and treated for 5 d with indicated drug concentrations. The percentage of apoptotic cells was measured as described in Materials and Methods. The data are presented as a boxplot diagram along with the corresponding P-values for 500 nM-MLN-points (Wilcoxon test).

Discussion-Supplementary Figure S12. Recent studies have shown that CRL inhibition by MLN induces autophagy that protects cancer cells from apoptosis; moreover, blocking autophagy markedly enhanced drug efficacy (Zhao et al, 2012). However in our case, examination of autophagy markers revealed that, although MLN increased the level of the lipidated form of LC3, it also resulted in an increa se, but not a degradation, of autophagy substrate p62/SQSTM1 (Supplementary Figure S12A). The la tter suggests that the effect of MLN on autophagy is complex and may include a block of the autopha gosomes at the terminal stages (Klionsky et al, 2016). This is also corroborated by the measurements of autophagic flux (Klionsky et al, 2016), which showed no clear effect of MLN on autophagy markers in the presence of Bafilomycin A1, an inhibitor of lysosomal degradation (Supplementary Figure S12B). Specifically, a similar MLN-Bafilomycin A1 interaction was also observed by Zhao *et al.* (Zhao et al, 2012). Finally, autophagy inhibitors did not affect significantly VCaP apoptotic response to MLN, thus excluding the major role of autophagy stimulation in the observed MLN phenotypes (Supplementary Figure S12C).

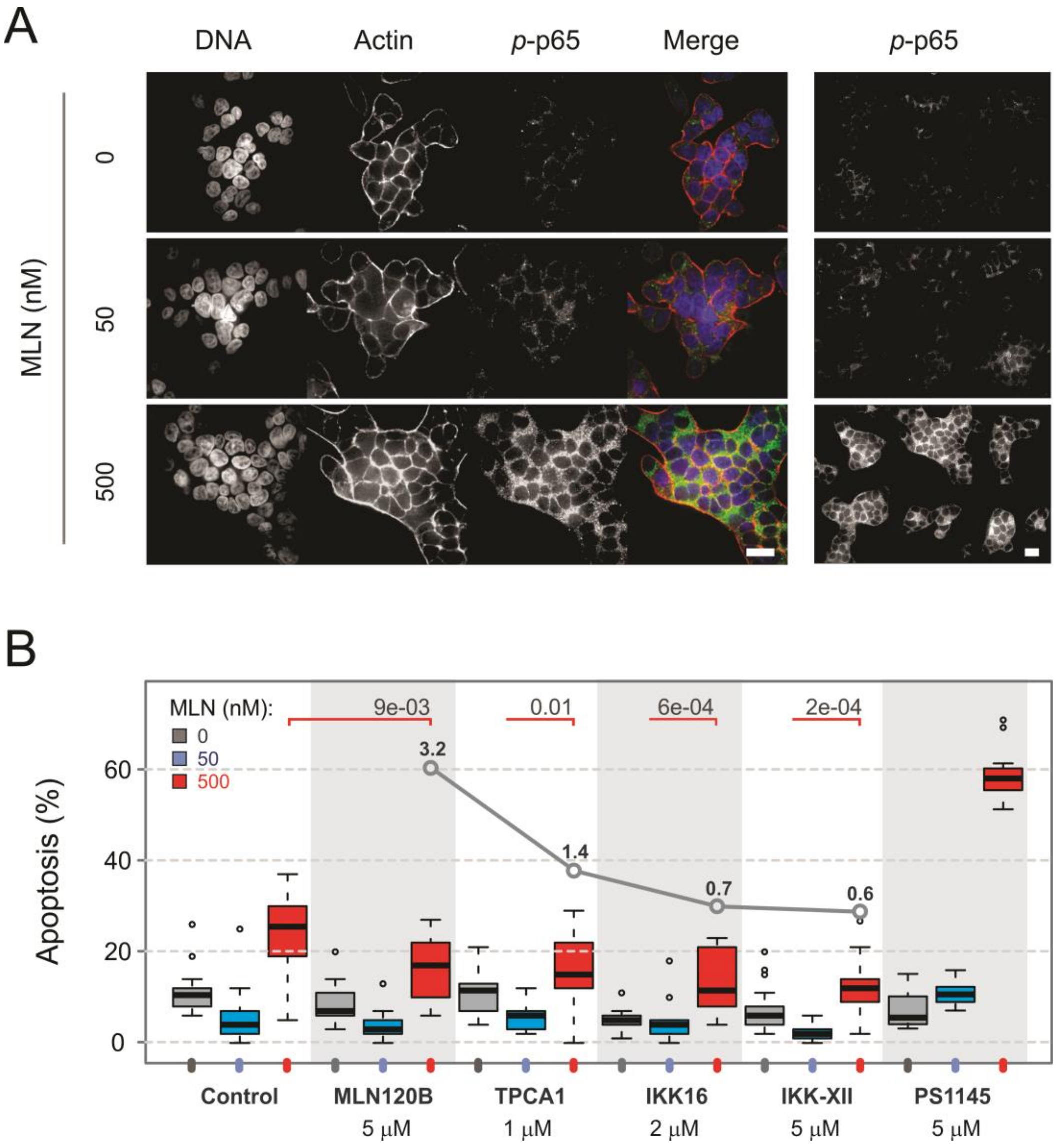

Supplementary Figure S13. MLN interaction with NFkB pathway. A, MLN induces accumulation of *p*-p65 in cytoplasmic speckles. Immunofluorescence analysis of *p*-p65 (phospho- Ser536) in VCaP cells treated with indicated concentrations of MLN. Cells were probed with *p*-p65-specific antibody (*p*-p65, green), actin-specific antibody (Actin, red), and Hoechst dye (DNA, blue). Scale bar is 20 µm. B, Effect of IKK inhibitors on MLN-induced apoptosis. Cells were treated for 5 d with indicated drug concentrations in 10% StdM. The percentage of apoptotic cells was measured as described in Materials and Methods. The data are presented as a boxplot diagram along with the corresponding P-values for 500 nM–MLN-points (Wilcoxon test). The four-point plot inside the diagram shows IKKβ-to-IKKα selectivity of the drugs (Tian et al, 2015) (see also Supplementary Table S5).

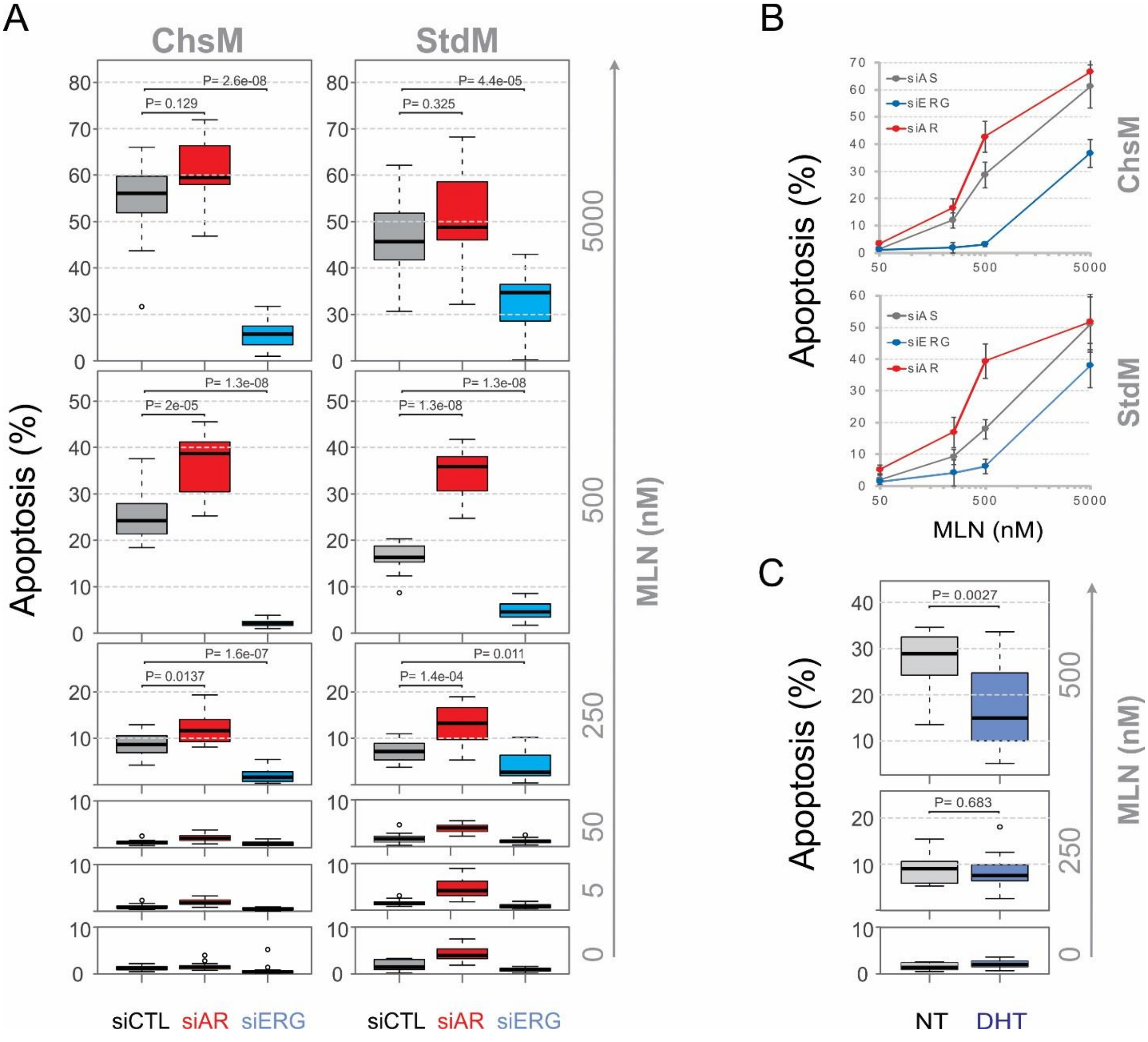

Supplementary Figure S14. Opposite roles of AR and ERG in MLN-induced cell fate. A, Opposite effects of AR and ERG knockdowns on MLN-induced apoptosis. VCaP cells grown in 10% ChSM or 10% StdM were transfected with indicated siRNAs and treated with MLN on the next day. The percentage of apoptotic cells was measured as described in Materials and Methods. The data are shown as a boxplot diagram along with the corresponding P-values (Wilcoxon test, see Materials and Methods for more details). B, The same data presented as line charts (mean ± s.d.). C, Effect of dihydrotestosterone (DHT) on MLN-induced apoptosis. VCaP cells grown in 10% ChSM with (DHT) or without (NT) 1 nM DHT were treated with MLN for 5 days. The percentage of apoptotic cells was measured as described in Materials and Methods. The data were analyzed as described above.

SUPPLEMENTARY TABLES

Protein	Manufacturer	Reference	Application
Androgen Receptor	Thermo Scientific	MA5-13426	WB
β-catenin (total)	Transduc lab	#610468	WB/IF
β-catenin (phospho-Ser33/37/Thr41)	Cell Signaling	#9561	WB/IF
c-Myc	Santa Cruz	(1.N.2): sc-70469	WB
CAND1	Abnova	H00055832	WB
Cdt1	Abcam	ab70829	WB
ERG	Abcam	[EPR3864] (ab92513)	WB
FKBP51	Santa Cruz	(H100) : sc-13983	WB
Frizzled-4	Santa Cruz	(C-18): sc-66450	WB
GAPDH	Santa Cruz	sc-25778	WB
γH2AX (phospho-Ser139)	Millipore	16-202A	WB/IF
IκB	Cell Signaling	#4814	WB
IκB (phospho-Ser32)	Cell Signaling	#2859	WB
LEF1	Millipore	17-604	WB
NEDD8	Abcam	ab81264	WB/IF
NF-κB p65	Cell Signaling	#8242	WB/IF
NF-κB (phospho-Ser536)	Cell Signaling	#3033	WB/IF
P21	Santa Cruz	(C-19): sc-397	WB
P27	Cell Signaling	#3686	WB
P53	Santa Cruz	(FL-393 G): sc-6243-G	WB
PSA	DAKO	A0562	WB
RBX1	Millipore	AB3737	WB
SLC45A3	Santa Cruz	(A-5) : sc-393069	WB
SUMO-1	Santa Cruz	(FL-101): sc-9060	WB
Ubiquitin	Santa Cruz	(P4D1): sc-8017	WB

Supplementary Table S1. List of the antibodies.

Gene Symbol	Smart Pool® Catalog Number	Duplex Catalog Number	GENEID	Gene Accession	GINumber	Sequence	Figure 4
**CACUL1**	L-016305-01	J-016305-09	143384	NM_153810	54262140	AGGCGAUGAUGGACGACCA	
**CACUL1**	L-016305-01	J-016305-10	143384	NM_153810	54262140	GGAUAUUGGGAGCAAGUAA	
**CACUL1**	L-016305-01	J-016305-11	143384	NM_153810	54262140	GGGUACAGAUAGUGAAUGU	
**CACUL1**	L-016305-01	J-016305-12	143384	NM_153810	54262140	GCAUUAGAAAGUCUUGUUA	
**CAND1**	L-015562-01	J-015562-09	55832	NM_018448	21361793	GACUUUAGGUUUAUGGCUA	siCAND1-1
**CAND1**	L-015562-01	J-015562-10	55832	NM_018448	21361793	CGUGCAACAUGUACAACUA	siCAND1-2
**CAND1**	L-015562-01	J-015562-11	55832	NM_018448	21361793	CAACAAGAACCUACAUACA	
**CAND1**	L-015562-01	J-015562-12	55832	NM_018448	21361793	CAUAACAAGCCAUCAUUAA	
**CAND2**	L-023448-01	J-023448-09	23066	XM_944849	88968671	ACGAGGACAGCGAGCGCAA	
**CAND2**	L-023448-01	J-023448-10	23066	XM_944849	88968671	GCACCCUGAUCCAAUGUUU	
**CAND2**	L-023448-01	J-023448-11	23066	XM_944849	88968671	AGAACGGUGAGGUGCAGAA	
**CAND2**	L-023448-01	J-023448-12	23066	XM_944849	88968671	UGUCGGAGUUGCAGAAGGA	
**CDC34**	L-003230-00	J-003230-13	997	NM_004359	16357476	GCUCAGACCUCUUCUACGA	
**CDC34**	L-003230-00	J-003230-14	997	NM_004359	16357476	GGACCAUUCUCCUGAGUGU	
**CDC34**	L-003230-00	J-003230-15	997	NM_004359	16357476	CCUGACACCACCAGAAUAA	
**CDC34**	L-003230-00	J-003230-16	997	NM_004359	16357476	GAUCGGGAGUACACAGACA	
**CUL1**	L-004086-00	J-004086-06	8454	NM_003592	32307160	CAACGAAGAGUUCAGGUUU	
**CUL1**	L-004086-00	J-004086-07	8454	NM_003592	32307160	CGAGGAAGACCGCAAACUA	
**CUL1**	L-004086-00	J-004086-08	8454	NM_003592	32307160	AGACAGUGCUUGAUGUUCA	
**CUL1**	L-004086-00	J-004086-09	8454	NM_003592	32307160	CAUAGAAGACAAAGACGUA	
**CUL2**	L-007277-00	J-007277-05	8453	NM_003591	19482173	GGAAGUGCAUGGUAAAUUU	
**CUL2**	L-007277-00	J-007277-06	8453	NM_003591	19482173	CAUCCAAGUUCAUAUACUA	
**CUL2**	L-007277-00	J-007277-07	8453	NM_003591	19482173	GCAGAAAGACACACCACAA	
**CUL2**	L-007277-00	J-007277-08	8453	NM_003591	19482173	UGGUUUACCUCAUAUGAUU	
**CUL3**	L-010224-00	J-010224-06	8452	NM_003590	45827792	GAAGGAAUGUUUAGGGAUA	
**CUL3**	L-010224-00	J-010224-07	8452	NM_003590	45827792	GAGAUCAAGUUGUACGUUA	
**CUL3**	L-010224-00	J-010224-08	8452	NM_003590	45827792	GAAAGUAGACGACGACAGA	
**CUL3**	L-010224-00	J-010224-09	8452	NM_003590	45827792	GCACAUGAAGACUAUAGUA	
**CUL4A**	L-012610-00	J-012610-05	8451	NM_003589	57165422	GCACAGAUCCUUCCGUUUA	
**CUL4A**	L-012610-00	J-012610-06	8451	NM_003589	57165422	GAACAGCGAUCGUAAUCAA	
**CUL4A**	L-012610-00	J-012610-07	8451	NM_003589	57165422	GCAUGUGGAUUCAAAGUUA	
**CUL4A**	L-012610-00	J-012610-08	8451	NM_003589	57165422	GCGAGUACAUCAAGACUUU	
**CUL4B**	L-017965-00	J-017965-05	8450	NM_003588	28372492	UAAAUAACCUCCUUGAUGA	
**CUL4B**	L-017965-00	J-017965-06	8450	NM_003588	28372492	CAGAAGUCAUUAAUUGCUA	
**CUL4B**	L-017965-00	J-017965-07	8450	NM_003588	28372492	CGGAAAGAGUGCAUCUGUA	
**CUL4B**	L-017965-00	J-017965-08	8450	NM_003588	28372492	GCUAUUGGCCGACAUAUGU	
**CUL5**	L-019553-00	J-019553-05	8065	NM_003478	67514034	GACACGACGUCUUAUAUUA	
**CUL5**	L-019553-00	J-019553-06	8065	NM_003478	67514034	GCAAAUAGAGUGGCUAAUA	
**CUL5**	L-019553-00	J-019553-07	8065	NM_003478	67514034	UAAACAAGCUUGCUAGAAU	

Gene Symbol	Smart Pool® Catalog Number	Duplex Catalog Number	GENEID	Gene Accession	GINumber	Sequence	Figure 4
**CUL5**	L-019553-00	J-019553-08	8065	NM_003478	67514034	CGUCUAAUCUGUUAAAGAA	
**CUL7**	L-017673-00	J-017673-05	9820	NM_014780	41872645	GAUCUUGGGCUUUGAGGAA	
**CUL7**	L-017673-00	J-017673-06	9820	NM_014780	41872645	CUAGUGAGGACUCGAGUUA	
**CUL7**	L-017673-00	J-017673-07	9820	NM_014780	41872645	GAACCUAGAUGGGGAGAUU	
**CUL7**	L-017673-00	J-017673-08	9820	NM_014780	41872645	GACGUGAAGUCCCUCAUUC	
**DCUN1D1**	L-019139-00	J-019139-05	54165	NM_020640	36030882	GGAUAAAGUUCGUCAGUUU	
**DCUN1D1**	L-019139-00	J-019139-06	54165	NM_020640	36030882	GCAUUAGUGUGUUGAUUAU	
**DCUN1D1**	L-019139-00	J-019139-07	54165	NM_020640	36030882	CCAGGACGAUUUAAGGAUU	
**DCUN1D1**	L-019139-00	J-019139-08	54165	NM_020640	36030882	GAACUUAGUGCUUAAUGGA	
**DCUN1D2**	L-020261-01	J-020261-09	55208	NM_001014283	62122951	GGGAGAGGAUCUUGUCGUA	
**DCUN1D2**	L-020261-01	J-020261-10	55208	NM_001014283	62122951	GCCAGCAAUUCACGAUUUA	
**DCUN1D2**	L-020261-01	J-020261-11	55208	NM_001014283	62122951	ACAGGGAGUCCAUGCGGAA	
**DCUN1D2**	L-020261-01	J-020261-12	55208	NM_001014283	62122951	CAUCAUAGCUUUUGCGUUA	
**DCUN1D3**	L-018390-00	J-018390-05	123879	NM_173475	27735046	AAGGAUCUCUACCGGUUUA	
**DCUN1D3**	L-018390-00	J-018390-06	123879	NM_173475	27735046	ACGGUUCCCUAGCCUCUUA	
**DCUN1D3**	L-018390-00	J-018390-07	123879	NM_173475	27735046	CCAGAACAAUCCUCCGGUA	
**DCUN1D3**	L-018390-00	J-018390-08	123879	NM_173475	27735046	GUAAGAAUCCCUCAUCGAC	
**DCUN1D4**	L-014118-01	J-014118-09	23142	NM_015115	32698693	GGUGACAUGUGAUCGUUUA	
**DCUN1D4**	L-014118-01	J-014118-10	23142	NM_015115	32698693	GUGCAAUGUCCUAGAGUUU	
**DCUN1D4**	L-014118-01	J-014118-11	23142	NM_015115	32698693	GAAUAUAGGUACCAAUGAA	
**DCUN1D4**	L-014118-01	J-014118-12	23142	NM_015115	32698693	CCAACUAUCUGGUGCUAUU	
**DCUN1D5**	L-014842-01	J-014842-09	84259	NM_032299	34147410	CAAUCAAAGUAUCGUGUUA	
**DCUN1D5**	L-014842-01	J-014842-10	84259	NM_032299	34147410	GUUGAAUGAUAUUUCGUCA	
**DCUN1D5**	L-014842-01	J-014842-11	84259	NM_032299	34147410	CCGUCAGACAUCAUAGCAA	
**DCUN1D5**	L-014842-01	J-014842-12	84259	NM_032299	34147410	UGAUGGGCAUUGAGCCACA	
**NAE1**	L-006401-00	J-006401-05	8883	NM_001018160	66363687	GAUGAUCGCUGCAUAAAUA	
**NAE1**	L-006401-00	J-006401-06	8883	NM_001018160	66363687	GCACAGUGGUAUAGUGAAA	
**NAE1**	L-006401-00	J-006401-07	8883	NM_001018160	66363687	GAUUUUAGCUCGUGCCUUA	
**NAE1**	L-006401-00	J-006401-08	8883	NM_001018160	66363687	GUUACGGGCUGUUGAUAGA	
**RBX1**	J-004087-00	J-004087-07	9978	NM_014248	22091459	GAAGCGCUUUGAAGUGAAA	
**RBX1**	J-004087-00	J-004087-08	9978	NM_014248	22091459	GGGAUAUUGUGGUUGAUAA	
**RBX1**	J-004087-00	J-004087-09	9978	NM_014248	22091459	GGAACCACAUUAUGGAUCU	siRBX1-1
**RBX1**	J-004087-00	J-004087-10	9978	NM_014248	22091459	CAUAGAAUGUCAAGCUAAC	siRBX1-2
**SAG**	J-011105-00	J-011105-08	6295	NM_000541	10880124	GCAAGAGAGCCUGCUUAAA	
**SAG**	J-011105-00	J-011105-06	6295	NM_000541	10880124	GUUCUCUACUCGAGUGAUU	
**SAG**	J-011105-00	J-011105-07	6295	NM_000541	10880124	GAACCGAACCAUGUUAUCU	
**SAG**	J-011105-00	J-011105-05	6295	NM_000541	10880124	AAAGUUAUCAGGAUGCAAA	
**UBA3**	L-005249-00	J-005249-05	9039	NM_198197	38045945	CAAUAGUGCUUCUCUGCAA	
**UBA3**	L- 005249-00	J-005249-06	9039	NM_198197	38045945	UACAGGAGGUUUUGGAUUA	

Gene Symbol	Smart Pool® Catalog Number	Duplex Catalog Number	GENEID	Gene Accession	GINumber	Sequence	Figure 4
**UBA3**	L-005249-00	J-005249-07	9039	NM_198197	38045945	GAUAAAUGGCAUGCUGAUA	
**UBA3**	L-005249-00	J-005249-08	9039	NM_198197	38045945	CAAUCUAAAUAGGCAGUUU	
**UBE2F**	L-009081-01	J-009081-09	140739	NM_080678	18087856	CAAGUAAACUGAAGCGUGA	
**UBE2F**	L-009081-01	J-009081-10	140739	NM_080678	18087856	AUGACUACAUCAAACGUUA	
**UBE2F**	L-009081-01	J-009081-11	140739	NM_080678	18087856	CAAUAAGAUACCCGCUACA	
**UBE2F**	L-009081-01	J-009081-12	140739	NM_080678	18087856	CUGAAGUUCCCGAUGCGUA	
**UBE2M**	L-004348-00	J-004348-05	9040	NM_003969	37577133	GAAAUAGGGUUGGCGCAUA	
**UBE2M**	L-004348-00	J-004348-06	9040	NM_003969	37577133	AAGCCAGUCCUUACGAUAA	
**UBE2M**	L-004348-00	J-004348-07	9040	NM_003969	37577133	UUAAGGUGGGCCAGGGUUA	
**UBE2M**	L-004348-00	J-004348-08	9040	NM_003969	37577133	GAUGAGGGCUUCUACAAGA	
**AR**	J-003400-00	J-003400-05	367	NM_001011645	58535454	GAGCGUGGACUUUCCGGAA	
**AR**	J-003400-00	J-003400-06	367	NM_001011645	58535454	UCAAGGAACUCGAUCGUAU	
**AR**	J-003400-00	J-003400-07	367	NM_001011645	58535454	CGAGAGAGCUGCAUCAGUU	

Supplementary Table S2. List of siRNAs.

Acronym	Luciferase reporter	Response elements	Pathway	Reference
**3-5M**	3' +5' Myc	5' plus 3' c-MYC enhancer	Wnt/ b-Cat/ others	(Yochum et al, 2010)
**ARE**	TAT-GRE-EIB	androgen/glucocorticoid RE	AR/ GR	(Jenster et al, 1991)
**ATF6**	5xATF6	ATF6 binding site	ER-stress	(Wang et al, 2000)
**CSL**	4xCSL	RBP-Jk binding site	Notch	(Ann et al, 2011)
**CTL**	pGL3-control	SV40 promoter	Firefly-control	Promega
**DBE**	3xDBE	FoxO binding site	FoxO	(Zanella et al, 2009)
**DBE***	3xDBE*	FoxO mut-binding site	FoxO-control	(Zanella et al, 2009)
**EBS**	6xETS	ETS binding site	ERG	(Nhili et al, 2013)
**EGR1**	EGR1	EGR1 promoter	c-Myc/ others	(Zhang et al, 2013)
**EMS**	4xEMS	E-box Myc sequence	c-Myc	(Zhang et al, 2013)
**FOS**	FOS	c-fos promoter	MAPK/cAMP/others	(Cen et al, 2003)
**GLI**	8xGli	Gli binding site	Hedgehog	ATCC
**HES1**	HES1	HES1 promoter	Notch/ others	(Jarriault et al, 1995)
**HRE**	3xHRE	hypoxia response element	Hypoxia/ FoxO/ others	(Emerling et al, 2008)
**kB3**	κB3	NF-κB binding site	NF-κB	(Deng et al, 2000)
**DRL**	LDLR	LDLR promoter (LDLRp)	Akt/ mTOR/ others	(Shimano et al, 1997)
**NFAT**	3xNFAT/AP1	NFAT/AP1 binding site	NFAT/AP1	(Macián et al, 2000)
**PAI1**	PAI1	PAI1 promoter	TGF-β/ others	(Abe et al, 1994)
**PYE**	4xPye	Py enhancer element	ERG	(Carrère et al, 1998)
**RLU**	pRL-TK	TK promoter	Renilla-control	Promega
**SBE**	12xSBE	Smad-binding element	TGF-β/ others	(Dennler et al, 1998)
**SRE**	3x(2-3LDLR)	repeats 2 and 3 of the LDLRp	Akt/ mTOR/ others	(Shimano et al, 1997)
**STF**	14xSTF	super TOP-flash, TCF/ Lef1	Wnt/β-Cat	(Lee et al, 2013)

Supplementary Table S3. List of reporter plasmids.

Gene	Direction	Sequence (5' → 3')
PSA	Forward	GATGAAACAGGCTGTGCCG
	Reverse	CCTCACAGCTGCCCACTGCA
TMPRSS2:ERG	Forward	GAGCGCCGCCTGGAG
	Reverse	TAGGCACACTCAAACAACGACTG
18S	Forward	CGATGCGCCGGCGTTATT
	Reverse	CCTGGTGGTGCCCTTCCGT
SLC45A3	Forward	CGCCATCTCCCTGGTCTT
	Reverse	CAGTGTCCCCTCGGTATTTG
FKBP1	Forward	AAAAGGCCAAGGAGCACAAC
	Reverse	TTGAGGAGGGGCCGAGTTC

Supplementary Table S4. List of qPCR primers.

Drug	Target	MW	IC50 (nM)	Concentration (nM)	Source
MLN4924	NAE	444	5	0-5000	Interchim
Olaparib	PARP1	434	5	1000	Adooq
NU7441	DNA-PK	413	14	1000	ApexBio
MK1775	Wee1	500	5	1000	Adooq
BI2536	Plk1	522	1	10	MedChemExpress
JQ1	Brd4/BET	457	77	100	ApexBio
Etoposide	TopIIb	589	120-600[1]	1000	Sigma-Aldrich
iCRT3	β-Cat	395	8	5000	Sigma-Aldrich
XAV939	TNKS	312	10	1000	Santa Cruz Biotech
PS1145	IKK	396	100(N.R.)[2]	5000	Santa Cruz Biotech
TPCA1	IKK	279	18(400)[2]	1000	MedChemExpress
MLN120B	IKK	367	60(100000)[2]	5000	MedChemExpress
IKK16	IKK	484	40(200)[2]	2000	Adooq
IKK-XII	IKK	402	100(400)[2]	5000	Merk Chemicals

Supplementary Table S5. List of chemical inhibitors.
[1] IC50 for cell growth inhibition in sensitive cell lines.
[2] Values are given for IKKβ, and for IKKα in parenthesis. N.R.- not reported.

SUPPLEMENTARY REFERENCES

Abe M, Harpel J, Metz C, Nunes I, Loskutoff D, Rifkin D (1994) An assay for transforming growth factor-β using cells transfected with a plasminogen activator inhibitor-1 promoter-luciferase construct. *Analytical biochemistry* **216:** 276-284

Ann EJ, Kim HY, Choi YH, Kim MY, Mo JS, Jung J, Yoon JH, Kim SM, Moon JS, Seo MS (2011) Inhibition of Notch1 signaling by Runx2 during osteoblast differentiation. *Journal of Bone and Mineral Research* **26:** 317-330

Birmingham A, Selfors LM, Forster T, Wrobel D, Kennedy CJ, Shanks E, Santoyo-Lopez J, Dunican DJ, Long A, Kelleher D (2009) Statistical methods for analysis of high-throughput RNA interference screens. *Nature methods* **6:** 569-575

Carrère S, Verger A, Flourens A, Stehelin D, Duterque-Coquillaud M (1998) Erg proteins, transcription factors of the Ets family, form homo, heterodimers and ternary complexes via two distinct domains. *Oncogene* **16**

Cen B, Selvaraj A, Burgess RC, Hitzler JK, Ma Z, Morris SW, Prywes R (2003) Megakaryoblastic leukemia 1, a potent transcriptional coactivator for serum response factor (SRF), is required for serum induction of SRF target genes. *Molecular and cellular biology* **23:** 6597-6608

Debacq-Chainiaux F, Erusalimsky JD, Campisi J, Toussaint O (2009) Protocols to detect senescence-associated beta-galactosidase (SA-betagal) activity, a biomarker of senescent cells in culture and in vivo. *Nature protocols* **4:** 1798-1806

Deng L, Wang C, Spencer E, Yang L, Braun A, You J, Slaughter C, Pickart C, Chen ZJ (2000) Activation of the IκB kinase complex by TRAF6 requires a dimeric ubiquitin-conjugating enzyme complex and a unique polyubiquitin chain. *Cell* **103:** 351-361

Dennler S, Itoh S, Vivien D, ten Dijke P, Huet S, Gauthier JM (1998) Direct binding of Smad3 and Smad4 to critical TGFβ-inducible elements in the promoter of human plasminogen activator inhibitor-type 1 gene. *The EMBO journal* **17:** 3091-3100

Emerling BM, Weinberg F, Liu J-L, Mak TW, Chandel NS (2008) PTEN regulates p300-dependent hypoxia-inducible factor 1 transcriptional activity through Forkhead transcription factor 3a (FOXO3a). *Proceedings of the National Academy of Sciences* **105:** 2622-2627

Jarriault S, Brou C, Logeat F, Schroeter EH, Kopan R, Israel A (1995) Signalling downstream of activated mammalian Notch. *Nature* **377:** 355-358

Jenster G, van der Korput HA, van Vroonhoven C, van der Kwast TH, Trapman J, Brinkmann AO (1991) Domains of the human androgen receptor involved in steroid binding, transcriptional activation, and subcellular localization. *Molecular endocrinology* **5:** 1396-1404

Klionsky DJ, Abdelmohsen K, Abe A, Abedin MJ, Abeliovich H, Acevedo Arozena A, Adachi H, Adams CM, Adams PD, Adeli K (2016) Guidelines for the use and interpretation of assays for monitoring autophagy. *Autophagy* **12:** 1-222

Lee E, Madar A, David G, Garabedian MJ, DasGupta R, Logan SK (2013) Inhibition of androgen receptor and β-catenin activity in prostate cancer. *Proceedings of the National Academy of Sciences* **110:** 15710-15715

Lee YG, Korenchuk S, Lehr J, Whitney S, Vessela R, Pienta KJ (2001) Establishment and characterization of a new human prostatic cancer cell line: DuCaP. *In vivo* **15:** 157-162

Macián F, García-Rodríguez C, Rao A (2000) Gene expression elicited by NFAT in the presence or absence of cooperative recruitment of Fos and Jun. *The EMBO journal* **19:** 4783-4795

Nhili R, Peixoto P, Depauw S, Flajollet S, Dezitter X, Munde MM, Ismail MA, Kumar A, Farahat AA, Stephens CE (2013) Targeting the DNA-binding activity of the human ERG transcription factor using new heterocyclic dithiophene diamidines. *Nucleic acids research* **41:** 125-138

Shimano H, Horton JD, Shimomura I, Hammer RE, Brown MS, Goldstein JL (1997) Isoform 1c of sterol regulatory element binding protein is less active than isoform 1a in livers of transgenic mice and in cultured cells. *Journal of Clinical Investigation* **99:** 846

Tan SH, Furusato B, Fang X, He F, Mohamed AA, Griner NB, Sood K, Saxena S, Katta S, Young D, Chen Y, Sreenath T, Petrovics G, Dobi A, McLeod DG, Sesterhenn IA, Saxena S, Srivastava S (2014) Evaluation of ERG responsive proteome in prostate cancer. *The Prostate* **74:** 70-89

Tian F, Zhou P, Kang W, Luo L, Fan X, Yan J, Liang H (2015) The small-molecule inhibitor selectivity between IKK α and IKK β kinases in NF-κ B signaling pathway. *Journal of Receptors and Signal Transduction* **35:** 307-318

Wang Y, Shen J, Arenzana N, Tirasophon W, Kaufman RJ, Prywes R (2000) Activation of ATF6 and an ATF6 DNA binding site by the endoplasmic reticulum stress response. *Journal of Biological Chemistry* **275:** 27013-27020

Yochum GS, Sherrick CM, MacPartlin M, Goodman RH (2010) A β-catenin/TCF-coordinated chromatin loop at MYC integrates 5' and 3' Wnt responsive enhancers. *Proceedings of the National Academy of Sciences* **107:** 145-150

Zanella F, Rosado A, Garcia B, Carnero A, Link W (2009) Using multiplexed regulation of luciferase activity and GFP translocation to screen for FOXO modulators. *BMC cell biology* **10:** 14

Zhang Q, Spears E, Boone DN, Li Z, Gregory MA, Hann SR (2013) Domain-specific c-Myc ubiquitylation controls c-Myc transcriptional and apoptotic activity. *Proceedings of the National Academy of Sciences* **110:** 978-983

Zhao Y, Xiong X, Jia L, Sun Y (2012) Targeting Cullin-RING ligases by MLN4924 induces autophagy via modulating the HIF1-REDD1-TSC1-mTORC1-DEPTOR axis. *Cell death & disease* **3:** e386
